# Targeting the Protein–Membrane Interface Enables Design of Long-Acting CFTR Potentiators

**DOI:** 10.1021/acschembio.5c00993

**Published:** 2026-04-28

**Authors:** Johannes Morstein, Jonathan Borowsky, Shenghui Hu, YooJin Sheen, Victoria Nisoli, Tzyh-Chang Hwang, Michael Grabe, Kevan M. Shokat

**Affiliations:** a Department of Cellular and Molecular Pharmacology and Howard Hughes Medical Institute, 8785 University of California, San Francisco, California 94158, United States; b Division of Chemistry and Chemical Engineering, 6469 California Institute of Technology, Pasadena, California 91125, United States; c Department of Pharmaceutical Chemistry and Cardiovascular Research Institute, University of California, San Francisco, California 94143, United States; d Dalton Cardiovascular Research Center, 14716 University of Missouri-Columbia, Columbia, Missouri 65211, United States

## Abstract

Many therapeutically relevant membrane proteins possess druggable sites at the lipid–protein interface, but principles guiding ligand design for these sites are not well-defined. CFTR potentiators, a clinically validated drug class for cystic fibrosis, offer a compelling model for exploring membrane-targeted design principles, as their efficacy depends on sustained intramembrane binding to prolong channel opening and chloride conductance. Here, we systematically modified the lipophilic substituent of the CFTR potentiator ABBV-974 and identified an analog that confers markedly increased functional residence time, as measured by delayed current decay after compound washout in patch-clamp assays. Kinetic analysis incorporating the physicochemical properties of the lipophilic substituents suggests that this increased kinetic stability may arise from an increased residence time in the cell membrane, consistent with qualitative results of molecular dynamics simulations. These results establish a structure–function link between membrane-facing ligand modifications and functional target engagement and potentially offer generalizable strategies for designing probe molecules and drugs that stably engage lipid-exposed binding sites on membrane proteins.

## Graphical abstract

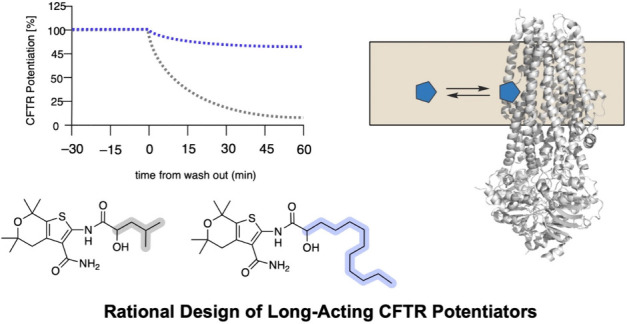

## Introduction

Integral membrane proteins such as ion channels, transporters, and GPCRs account for the majority of drug targets.^1^ While decades of medicinal chemistry have focused on optimizing ligand-protein interactions within well-defined aqueous-accessible binding pockets, an increasing number of pharmacophores are found to directly bind their targets at the protein–lipid interface.^2^ These intramembrane binding modes introduce distinct physicochemical and pharmacokinetic design challenges and opportunities not typically considered in standard drug discovery paradigms.^3,4^

CFTR potentiators offer a clinically validated and mechanistically tractable system to interrogate these principles.^5,6^ These compounds, such as ivacaftor and ABBV-974, bind to CFTR at an extrahelical lipid–protein interface to increase open probability and chloride transport in epithelial cells.^7,8^ Previous studies suggested that CFTR potentiators act as classical allosteric modulators: the open channel exhibits a stronger binding of the drug.^9^ Moreover, the measured steady-state affinity of the compound correlates inversely with the rate of current decay upon washout of the drug.^9,10^ Thus, functional effects are partly determined by target residence time, and rapid dissociation leads to a rapid loss of current.

In this study, we systematically modified the membrane-exposed site of ABBV-974 and assessed the resulting analogs in functional electrophysiology assays. We observed that specific lipophilic substitutions substantially delayed current decay after compound washout, suggesting prolonged target engagement. Molecular dynamics simulations are consistent with a scenario in which a lipophilic conjugate of ABBV-974 diffuses into the lipid bilayer upon unbinding from CFTR instead of sliding directly along the protein–membrane interface into water. Kinetic modeling shows that differences between current decay rates of ABBV-974 analogs can be predicted from differences in the logP of the hydrophobic substituents, suggesting that lipophilic substituents slow current decay by delaying dissociation from the membrane, enabling the potentiator to rebind to CFTR. Together, our data demonstrate that membrane-facing ligand modifications can enhance the functional residence time of CFTR potentiators, providing a potential rational framework for designing drugs that exploit the protein–lipid interface for improved kinetic stability.

## Results and Discussion

### Design and Synthesis of ABBV-974 Analogs Targeting the Protein–Membrane Interface

To investigate how ligand-membrane interactions influence functional engagement with CFTR, we designed a series of ABBV-974 analogs with systematic modifications to the membrane-exposed site of the molecule. Building on structural insights from the ABBV-974 bound CFTR structure (PDB: 6O1V; Figure 1B), we focused on modifying the site of pyrazole which extends into the membrane phase. For synthetic tractability we decided to swap the pyrazole for an alpha-hydroxy amide as similar fragments were found equipotent in the initial medicinal chemistry campaign.^6^ We synthesized a focused analog series, **CFTRi-C3**, **CFTRi-C6**, and **CFTRi-C10**, featuring linear alkyl chains of increasing length (isobutyl, hexyl, and decyl, respectively) as lipophilic substituents (Figure 2A,C). These modifications were designed to increase interactions with the surrounding lipid environment and hydrophobic protein residues without altering the core pharmacophore responsible for CFTR potentiation. The analogs were prepared via a concise synthetic route (Figure 2B), enabling late-stage diversification of the terminal lipophilic substituent. We also synthesized a fluorescently labeled analog, **CFTRi-NBD**, by introducing a nitrobenzoxadiazole (NBD) moiety at the same position. This probe was used for confocal cell imaging to assess if this ligand class exhibits strong interactions with the plasma membrane.

**Figure 1:**
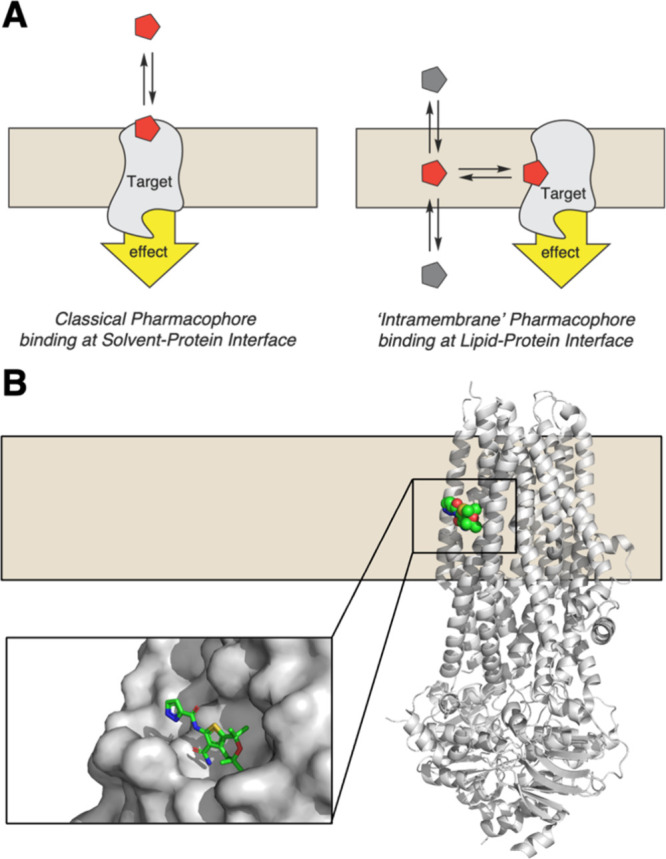
Ligand binding at the protein–membrane interface exemplified by CFTR potentiators. (A) Schematic comparison of classical drug binding to a solvent-accessible pocket (left) versus membrane access to an intramembrane binding site (right). (B) Cryo-EM structure of human CFTR bound to the potentiator ABBV-974 (PDB: 6O1V^7^), showing the drug bound at an extrahelical site exposed to the membrane.

**Figure 2:**
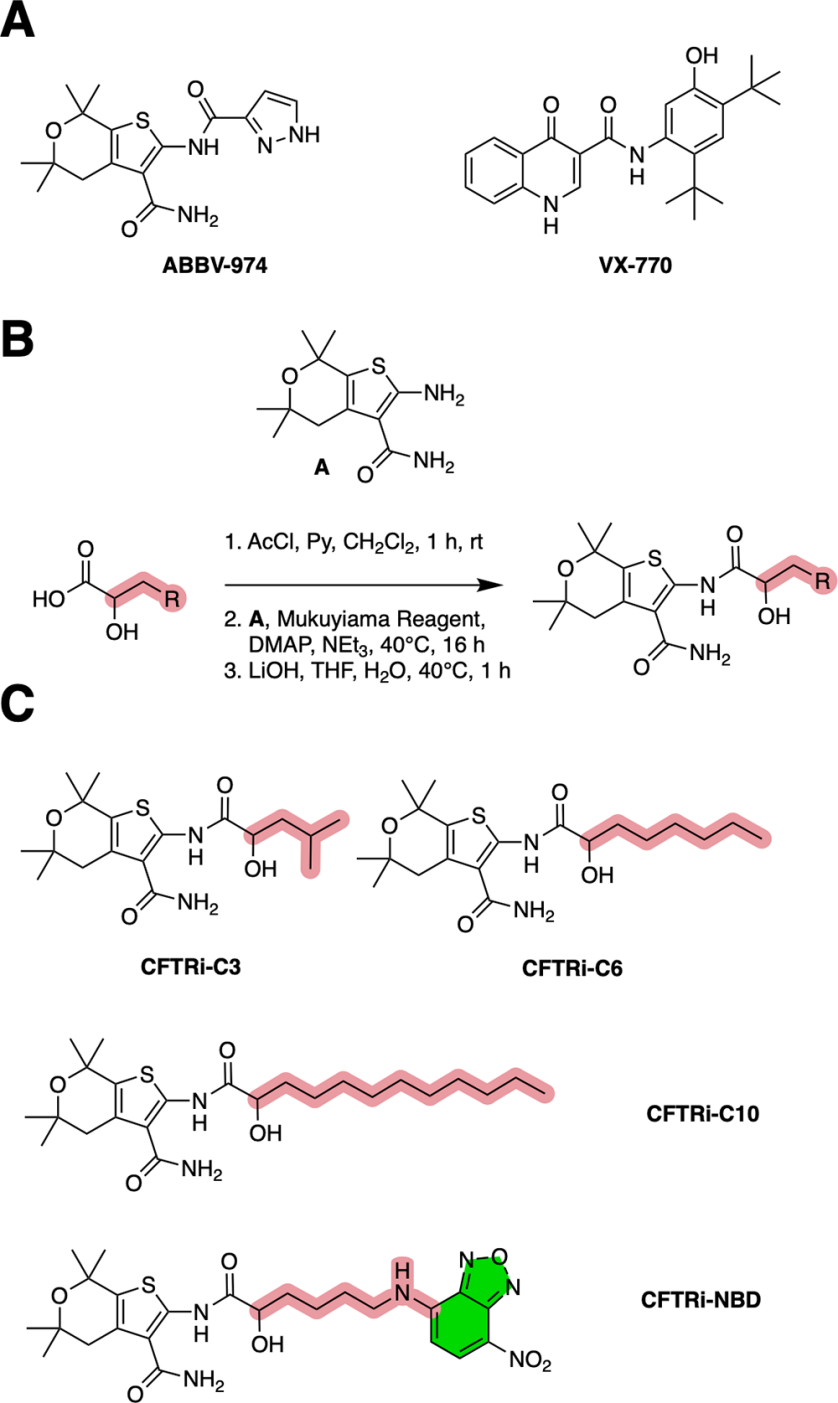
Chemical structures and synthetic strategy for CFTR potentiator analogs. (A) Structures of the CFTR potentiators ABBV-974 and ivacaftor. (B) General synthetic route used to generate analogs with modified lipophilic moieties at the membrane-exposed region of ABBV-974. (C) Representative structures of synthesized analogs **CFTRi-C3**, **CFTRi-C6**, **CFTRi-C10**, and **CFTRi-NBD**, highlighting systematic variation in the lipophilic side chain and incorporation of a fluorescent reporter group.

### Live Cell Imaging and Electrophysiology with ABBV-974 Analogs

To explore how structural modifications of ABBV-974 influence subcellular localization and functional engagement with CFTR, we employed live-cell confocal microscopy and electrophysiology. Live-cell imaging of the fluorescently labeled analog **CFTRi-NBD** revealed strong colocalization with ER-tracker (Pearson’s R = 0.84) and minimal overlap with a plasma membrane marker (Pearson’s R = −0.42), indicating that the compound preferentially accumulates in the endoplasmic reticulum rather than the plasma membrane (Figure 3A). This result suggests that the ABBV-974 scaffold may intrinsically favor intracellular membranes, which could limit its ability to engage plasma membrane-localized CFTR channels in a sustained manner. The ER accumulation of **CFTRi-NBD** offers a plausible explanation for the rapid off-rate observed with ABBV-974, as effective target binding would require high local concentrations of drug at the plasma membrane. To assess how structural modifications influence functional target engagement, we performed patch-clamp recordings in CHO cells transiently expressing CFTR. CFTR channels in excised inside-out membrane patches were activated with protein kinase A (PKA) and ATP until a steady-state current level was attained. The membrane patch was then treated with each analog in the continuous presence of ATP, followed by washout with drug-free solution, and CFTR-mediated chloride currents were monitored over time. ABBV-974 and the isobutyl analog **CFTRi-C3** induced robust chloride currents (potentiation 2.94 ± 0.91; n = 7) that rapidly declined upon washout (Figure 3B), consistent with fast dissociation and limited membrane anchoring. In contrast, **CFTRi-C6**, which features a hexyl chain (potentiation 1.74 ± 0.26; n = 3), showed a slower decay in current (Figure 3C), and the decyl analog **CFTRi-C10** (potentiation 2.52 ± 0.71; n = 4), exhibited prolonged current persistence postwashout (Figure 3D). These data indicate that increasing lipophilic chain length enhances functional residence time on CFTR.

**Figure 3:**
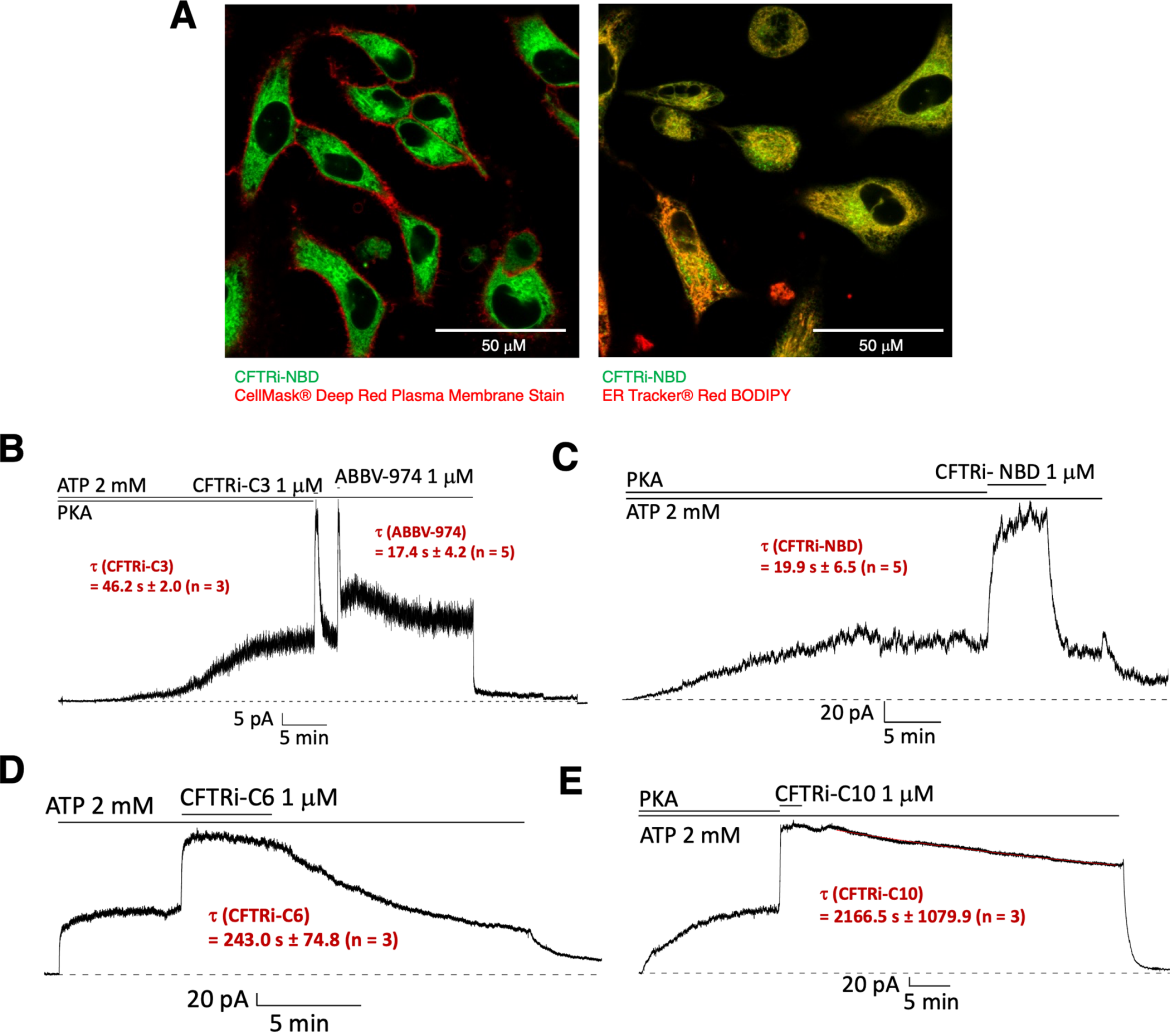
Subcellular localization and functional engagement of CFTR potentiator analogs. (A) Live-cell confocal microscopy of HeLa cells treated with **CFTRi-NBD** (green), stained with ER tracker and a plasma membrane marker. The NBD-labeled analog shows strong colocalization with the ER, but not with the plasma membrane, suggesting intracellular accumulation. (B–E) Patch-clamp recordings in an inside-out mode showing CFTR-mediated chloride currents in response to the application and washout of ABBV-974 analogs. (B) **ABBV-974** and **CFTRi-C3** show similar activation and rapid current decay upon washout. (C) **CFTRi-NBD** shows rapid washout on a time scale comparable to the parent compound **ABBV-974**. (D) **CFTRi-C6** shows a slower decline in current, indicating increased functional residence time. (E) **CFTRi-C10** exhibits sustained current after washout, consistent with stabilized binding at the protein–membrane interface.

### MD Simulations of Ligand Dissociation

To study the mechanism underlying the observed prolonged residence time of **CFTRi-C10**, we subsequently performed molecular dynamics (MD) simulations to investigate the process of ligand unbinding from the intramembrane **ABBV-974** binding site and the effect of conjugating a lipophilic group. Simulations were initiated from the bound **ABBV-974** CFTR structure (PDB 6O1V)^7^ embedded in a POPC lipid bilayer (Table 1). There are no CFTR-bound structures of the analogs synthesized in our study, so the pyrazole ring of **ABBV-974** was replaced with a 1-hydroxy-undecyl group *in silico* in an arbitrary orientation away from the protein. Consistent with the molecule’s ability to potently activate CFTR, the tail does not sterically clash with the protein.

**Table 1:** System Compositions

component	number in ABBV-974 simulations	number in CFTRi-C10 simulations
CFTR protein (in 5 pieces due to unresolved unmodeled loops)	1	1
ABBV-974	1	0
CFTRi-C10 (S enantiomer)	0	1
cholesterol	1	0
POPC	289 (140 in intracellular leaflet, 149 in extracellular leaflet)	289 (140 in intracellular leaflet, 149 in extracellular leaflet)
TIP3P water	40260	40198
Na^+^	109	108
Cl^–^	128	127

Simulations employed the Weighted Ensemble (WE)^11^ adaptive sampling method to model the CFTR dissociation process. This approach simulates many replicates of each molecular system, selectively pruning or replicating them to improve sampling along a predefined progress coordinate. To avoid biasing the direction in which the ligand unbinds, the center-of-mass (COM) distance of each ligand from its bound pose was chosen as the progress coordinate. Two WE simulations of each molecule were performed, capturing a single full dissociation event from each simulation as well as another partial event. The data sets collected for **ABBV-974** and **CFTRi-C10** contained 18 and 16.5 μs of aggregate simulation time, respectively (Table 2).

**Table 2:** Weighted Ensemble Simulation Details

ligand	ID	aggregate time (μs)	molecular time (ns)	bin boundaries (Å)
ABBV-974	1	12.0	100	0, 0.25, 0.5, 0.75, 1, 1.25, 1.5, 1.75, 2, 2.25, 2.5, 2.75, 3, 3.25, 3.5, 3.75, 4, 4.25, 4.5, 4.75, 5, 6, 7, 8, 9, 10, 11, 12, 15, 16, 17, 18, 19, 20, 21, 22, 23, 24, 25, 25.5, 26, 26.5, 27, 28, 29, 30, 32, 34, 36, 38, 40
ABBV-974	2	6.1	50	0, 0.25, 0.5, 0.75, 1, 1.25, 1.5, 1.75, 2, 2.25, 2.5, 2.75, 3, 3.25, 3.5, 3.75, 4, 4.25, 4.5, 4.75, 5, 6, 7, 8, 9, 10, 11, 12, 15, 16, 17, 18, 19, 20, 21, 22, 23, 24, 25, 25.5, 26, 26.5, 27, 28, 29, 30, 32, 34, 36, 38, 40
CFTRi-C10	1	5.7	100	0, 1, 3, 5, 7, 9, 10, 11, 12, 15, 16, 17, 18, 19, 19.5, 20, 20.5, 21, 21.5, 22, 22.5, 23, 24, 25, 25.5, 26, 26.5, 27, 28, 29, 30, 32, 34, 36, 38, 40
CFTRi-C10	2	10.8	100	0, 1, 1.3, 1.5, 1.7, 2, 2.2, 2.4, 2.5, 2.75, 3, 3.25, 3.5, 3.75, 4, 4.25, 4.5, 4.75, 5, 5.25, 5.5, 5.75, 6, 6.5, 7, 8, 9, 10, 11, 12, 13, 14, 15, 16, 17, 18, 19, 20, 21, 22, 23, 24, 25, 25.5, 26, 26.5, 27, 28, 29, 30, 32, 34, 36, 38, 40, 41

Despite the size of these data sets, well-converged energy profiles could not be obtained, preventing us from determining if the events described here represent the true steady state ensemble of dissociation trajectories. Nonetheless, there were molecular features that remained consistent across the simulations, such as the pathways through which molecules disengaged from the site and specific protein residues involved in these transitions. Additionally, a significant number of water molecules entered the membrane to solvate the membrane-facing E873-R933 salt bridge as the ligands dissociate (Figure S1 right two columns). Water penetration may be a natural feature reflecting the unusual chemical environment created by the membrane-exposed charges E873 and R933, or it could arise only in the presence of **ABBV-974**-like potentiators, but we cannot rule out that it is the result of insufficient sampling of membrane degrees of freedom orthogonal to the progress coordinate, which need time to relax in the wake of ligand motion. The average overall membrane thickness and the box length in the plane of the membrane undergo changes less than 1 Å as a function of WE progress coordinate, comparable to typical fluctuations at all points. This indicates that ligand unbinding and any associated water penetration did not significantly affect the shape and stability of the membrane (Figure S2).

All three **ABBV-974** unbinding events began with the molecule sliding out of its binding pocket along the protein–membrane interface in the direction of the membrane-facing E873-R933 salt bridge (purple, cyan, to green snapshots for both full events in Figure 4A,B, Figure S1A,B, left column). The NH group of the pyrazole ring then formed a hydrogen bond with E873 (shown as spheres). **ABBV-974** then rotated away from the protein, orienting itself with the bicyclic ring system pointing outward into the membrane with the pyrazole still hydrogen bonded to E873 (magenta ABBV-974 pose). Figure S3A,B shows consistent hydrogen bonding to the protein for both dissociation trajectories throughout this period, an example of which is depicted in Videos S1 and S2. It then detached from this radial position and diffused away into the membrane, while in the third partial unbinding event, the molecule retained some contact with the protein close to the cytoplasmic leaflet (Figure 4C). All three **CFTRi-C10** dissociation pathways began with the bicyclic ring disengaging from the binding site and moving toward the E873-R933 salt bridge as observed for **ABBV-974** (Figure S1C,D). However, only the trajectory in Figure 4E showed hydrogen bonding to E873 via the alcohol on the lipid tail, which takes the place of the NH group on the pyrazole (Figure S3D, Videos S3 and S4). In both WE simulations, the lipid tail was highly flexible even when the bicyclic rings are bound in the experimental pose. Once dissociated, ABBV-974 resided preferentially at the protein–membrane interface, with its pyrazole and terminal amide exposed to water (Figure 4C and Figures S4A,B, and S5A,B). Meanwhile, dissociated CFTRi-C10 remained entirely within the membrane core in one WE run, and mainly within the core in the other (Figure 4F, Figures S4C,D, and S5C,D). This difference may reflect the latter molecule’s greater hydrophobicity, but it could also be the case that the true equilibrium orientation places the bicyclic rings in or near the headgroup region and that this has not been sampled for CFTRi-C10 because our progress coordinate was not designed to sample it.

**Figure 4:**
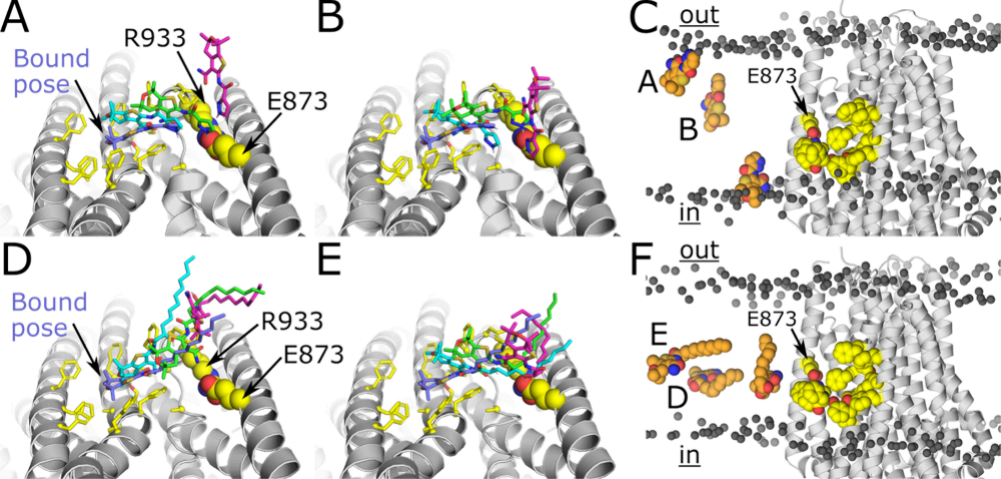
MD Simulations. (A, B) Two independent **ABBV-974** dissociation trajectories. The views are from the extracellular space. Bound **ABBV-974** from the equilibrated WE starting structure is shown in purple, followed by snapshots at later times in cyan, green, and magenta. Residues lining the binding site are shown in sticks, and the E873 and R933 are in van der Waals. (C) Final snapshots viewed from the membrane. Letters next to each molecule correspond to the trajectories from panels A and B, while the unlabeled molecule adjacent to the protein is the partial unbinding event. The protein is shown as gray ribbons, binding site residues as yellow spheres, and the phosphate groups on the lipids as dark gray spheres. R933 is hidden behind a phenylalanine ring directly below E873. (D, E) Two independent **CFTRi-C10** dissociation trajectories. (F) Final snapshots farthest from the binding site. Letters next to each molecule correspond to trajectories from panels D and E, and the unlabeled molecule corresponds to the partial unbinding event. Note that (D) and (E) come from WE runs with markedly different bin spacings. Views, numbering, and color scheme for panels (D–F) as in panels (A–C).

#### Hydrophobicity of Conjugated Lipophilic Group Predicts Washout Rates

Simulations suggest that ligands dissociate from the CFTR binding site into the membrane rather than slide along the protein–membrane interface into aqueous solution. A protein-independent membrane unbinding step is therefore assumed to be necessary for ligands to reach aqueous solution and wash out. A two-step model of this dissociation process was constructed based on this assumption to probe the relationships between different molecular interactions and current decay (Figure 5A). The current decay time constant T in a washout experiment is determined by three kinetic parameters: (1) k_–a_, the dissociation rate from the protein into the membrane, (2) *k*_a_, the association rate to the protein from the membrane, and (3) *k*_lw_, the lipid-to-water transition rate. As **ABBV-974** and its derivatives bind CFTR, k_–a_ < *k*_a_[P_t_], where [P_t_] is the total CFTR concentration, which is assumed to be constant between experiments, but the relative magnitudes of the other parameters are not obvious. There are two distinguishable kinetic regimes for the free molecules in the membrane, derived in the first section of the SI:

Scenario 1: Fast exit to the aqueous solution (*k*_lw_ ≫ *k*_a_[P_t_]). Upon dissociation from the protein, ligands quickly partition into aqueous solvent without rebinding. Thus, protein dissociation is rate limiting and the current decays exponentially with time constant
$$T=\frac{1}{k_{-a}}$$ 1
Scenario 2: Fast rebinding to the protein (*k*_a_[P_t_] ≫ *k*_lw_). Upon dissociation from the protein, rebinding dominates over wash out. The decay is not a true exponential but is well-approximated by a single exponential with time constant
$$T≅\frac{\left[P_{t}\right]}{1.53K_{d}k_{lw}}$$ 2
$$K_{d}=\frac{k_{-a}}{k_{a}}$$ 3

**Figure 5:**
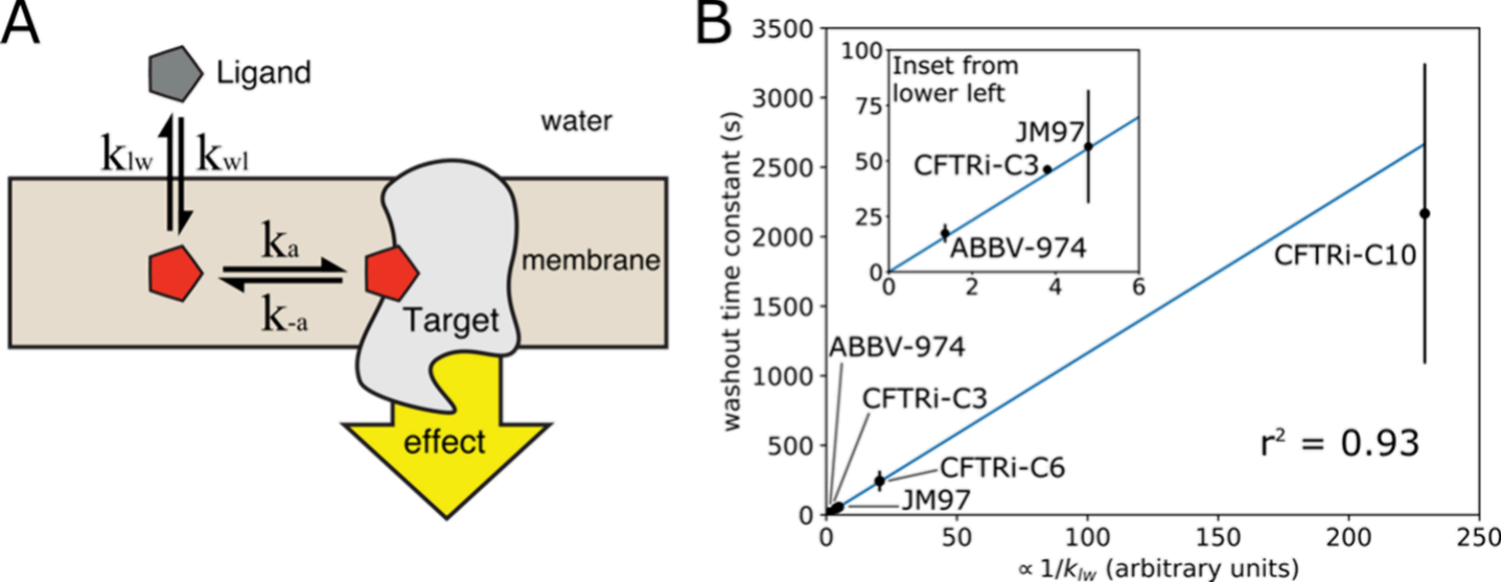
Kinetic model of potentiator washout. (A) A three-state kinetic model of ligand binding/unbinding to CFTR. *k*_a_ and k_–a_ are the rate constants of protein association and dissociation respectively, and *k*_wl_ and *k*_lw_ are the rate constants of membrane association and dissociation, respectively. (B) Dependence of washout rate on conjugated group logP, where the values on the *x* axis are the square roots of the experimental conjugated group partitioning coefficients P. The best fit line coefficient was obtained by minimizing squared errors in the ratios of actual to predicted time constants to avoid biasing the fit toward data points with larger absolute time constants. The r^2^ value was calculated with respect to the mean experimental rate for each analog. Error bars are 1 standard deviation.

Addressing scenario 2, the ratio of membrane dissociation rates *k*_lw_ for pairs of potentiator analogues can be estimated from experimentally available logP values of their respective R groups^12^ (see SI):

$$\frac{k_{lw1}}{k_{lw2}}=\frac{10^{-\log P_{1R}/2}}{10^{-\log P_{2R}/2}}$$ 4

As shown in Figure 5, these predicted dissociation rates are linearly proportional to the experimental washout rates to within one standard deviation, exactly as predicted by scenario 2 under the assumption that the protein–ligand affinity is R-group independent. The experimental rates would only be consistent with scenario 1 if the protein dissociation rate happened to be linearly proportional to the membrane dissociation rate, which is unlikely to be consistently true across structurally diverse R groups given the completely different structural and chemical details of the two transitions.

### Concluding Remarks

Our study demonstrates that introducing targeted lipophilic modifications to a CFTR potentiator scaffold can enhance functional drug-target residence time at the protein–membrane interface. Starting from **ABBV-974**, we synthesized a series of analogs with alkyl substituents of increasing chain length at a membrane-exposed vector. Electrophysiological recordings revealed that these modifications prolong CFTR activation following drug washout, consistent with increased kinetic stability. As seen in previous studies from our group a C10 medium chain lipid chain is particularly well-suited for this modulation and notably does not suffer a marked reduction in potency compared to the C6 tail. Medium chain lipid modifications are fairly common in natural products and are featured in a small number of FDA approved drugs.^13,14^ A kinetic model suggests that C6 and C10 substituents slow current decay following washout by slowing potentiator dissociation from the membrane, and provides a quantitative relation between the ratios of washout time constants and substituent logP values. Combined, our findings provide evidence that lipid exposed sites of pharmacophores are available for tuning to improve residence time of membrane-targeted probes and therapeutics and provide a modular design strategy to this end.

## Materials and Methods

### General Methods

Anhydrous solvents were purchased from Acros Organics. Unless specified below, all chemical reagents were purchased from Sigma-Aldrich, Oakwood, Ambeed, or Chemscene. Analytical thin layer chromatography (TLC) was performed using aluminum plates precoated with silica gel (0.25- mm, 60-Å pore size, 230–400 mesh, Merck KGA) impregnated with a fluorescent indicator (254 nm). TLC plates were visualized by exposure to ultraviolet light (UV). Flash column chromatography was performed with Teledyne ISCO CombiFlash EZ Prep chromatography system, employing prepacked silica gel cartridges (Teledyne ISCO RediSep). Proton nuclear magnetic resonance (^1^H NMR) spectra were recorded on Bruker AvanceIII HD instrument (400 MHz/100 MHz/376 MHz) at 23 °C operating with the Bruker Topspin 3.1. NMR spectra were processed using Mestrenova (version 14.1.2). Proton chemical shifts are expressed in parts per million (ppm, δ scale) and are referenced to residual protium in the NMR solvent (CHCl_3_: δ 7.26, MeOD: δ 3.31). Data are represented as follows: chemical shift, multiplicity (s = singlet, d = doublet, t = triplet, q = quartet, dd = doublet of doublets, dt = doublet of triplets, m = multiplet, br = broad, app = apparent), integration, and coupling constant (J) in Hertz (Hz). High-resolution mass spectra were obtained using a Waters Xevo G2-XS time-of-flight mass spectrometer operating with Waters MassLynx software (version 4.2). When LC-MS analysis of the reaction mixture is indicated in the procedure, it was performed as follows. An aliquot (1 μL) of the reaction mixture (or the organic phase of a mini-workup mixture) was diluted with 100 μL 1:1 acetonitrile/water. One μL of the diluted solution was injected onto a Waters Acquity UPLC BEH C18 1.7 μm column and eluted with a linear gradient of 5–95% acetonitrile/water (+0.1% formic acid) over 3.0 min. Chromatograms were recorded with a UV detector set at 254 nm and a time-of-flight mass spectrometer (Waters Xevo G2-XS).

### Confocal Imaging

HeLa cells were plated on a poly lysine precoated 8-well imaging dish at a density of 25k cells per well in DMEM/FBS/PS. After overnight incubation at 37 °C and 5% CO_2_, medium was removed and **CFTRi-NBD** (10 μM) and Organelle-Trackers (1:1000) were added medium for 15 min. Cells were washed with PBS and directly imaged on a Zeiss LSM880 inverted confocal microscope.

### Electrophysiology

Details of cell culture, transfection, and electrophysiological recordings can be found in previous reports.^9,10^ Briefly, patch-clamp electrophysiological recordings of CFTR channel currents were made in Chinese Hamster Ovary (CHO) cells transiently transfected with pcDNA plasmid containing the CFTR construct together with green fluorescence protein (GFP) cDNA (pEGFP-C3, Takara Bio, Shiga, Japan) using PolyFect transfection reagent (Qiagen, Hilden, Germany).

Patch-clamp electrodes were made from borosilicate capillary glass using a two-stage vertical puller (PP-81; Narishige, Amityville, NY). The pipet tips were fire polished with a homemade microforge to yield a pipet resistance of 2–4 MΩ when filled with a pipet solution containing (in mM): 140 NMDG-Cl (*N*-methyl-d-glucamine-chloride), 5 CaCl_2_, 2 MgCl_2_, and 10 HEPES, pH 7.4 with NMDG. The perfusing solution contained (in mM) 150 NMDG-Cl, 2 MgCl_2_, 10 EGTA, 8 Tris, and 10 HEPES, pH 7.4 with NMDG. The membrane patch was excised to an inside-out configuration with a seal resistance >40 GΩ. CFTR currents were recorded with an amplifier (EPC9; HEKA, Holliston, MA), filtered through an eight-pole Bessel filter (LPF-8; Warner Instruments, Hamden, CT) at 100 Hz and digitized to a computer at a sampling rate of 500 Hz with Pulse software (version 8.53; HEKA). The membrane potential was held at −30 mV; negative (inward) currents of CFTR were inverted for better visualization. Solution exchange was achieved by placing the pipet tip at the outlet of a fast solution change system (SF-77B; Warner Instruments). The patch was perfused with 25 IU (∼11 nM) PKA (Sigma-Aldrich P-2645) and 2 mM MgATP (Sigma-Aldrich) until the current reached a steady state before starting experiments. All experiments were performed at RT. All digitized data were quantified with Igor Pro 8.0 (WaveMetrics).

### System Construction for MD Simulations

Initial systems were built from the cryo-EM structure of ABBV-974-bound CFTR (PDB 6O1V) with the CFTR protein chain and ABBV-974. Cholesterol was retained for simulations of ABBV-974 only. All other ligands and the short non-CFTR protein chain from the experimental structure were removed from both systems. For CFTRi-C10, for which no experimental CFTR-bound structure is available, the pyrazole ring was deleted from the ABBV-974 structure and the C10 lipid tail was constructed in its place in an arbitrary orientation using PyMOL.^15^ Further model construction was performed using CHARMM-GUI 3.8.^16^ The structure was embedded in a POPC bilayer membrane and solvated in water and 150 mM NaCl using CHARMMM-GUI’s membrane modeler. Eleven waters were removed manually from the ABBV-974 system. Missing loops were left unmodeled due to their long length and distance from the ABBV-974 binding site. Residues next to missing stretches were capped with the appropriate acetyl and methylamine groups yielding neutral methyl amides. The system was parametrized by the Amber14SB protein force field for CFTR,^17^ the Lipid21 lipid force field for POPC and cholesterol,^18^ General Amber force field 2 for the potentiator,^19^ TIP3P water,^20^ and Joung-Cheatham ion parameters.^21^ See Table 1 for a list of components.

### MD Simulations

All MD simulations were run using GROMACS 2020.6.^22^ LINCS was used to constrain nonwater hydrogen atoms,^23^ and Particle Mesh Ewald was used to treat long-range electrostatics with a Fourier spacing of 0.12 nm.^24^ Thermostats were set to a temperature of 310.15 K, and barostats were set to a pressure of 1.01325 bar with a reference compressibility of 4.5 × 10^–5^ bar^–1^ and semi isotropic pressure coupling.

Equilibration: Each system was energy-minimized with harmonic restraints using the steepest descents algorithm, equilibrated in an NVT ensemble with harmonic restraints for 25 ps, and then equilibrated in an NPT ensemble for a total of 31 ns as the restraints were gradually removed. The Berendsen algorithm was used for the barostat and thermostat.^25^ Lennard-Jones forces were cut off at 0.9 nm during minimization and NVT steps using the cutoff scheme, and switched off between 1.0 and 1.2 nm using the force-switch scheme thereafter. The equilibration protocol is detailed in Table 3.

**Table 3:** Simulation Equilibration Protocol[Table-fn t3fn4]

equilibration step	simulation length (ps)	protein backbone, cholesterol, and ligand restraints	protein side chain restraints	POPC phosphorus restraints	dihedral restraints*	thermostat	barostat
1 (minimization; no dynamics)	n/a (5000 steps, emtol = 1000 kJ/(mol·nm))	4000	2000	1000	1000	n/a	n/a
2 (NVT)	25	4000	2000	1000	1000	Berendsen	none
3	25	4000	2000	1000	1000	Berendsen	Berendsen
4	25	2000	1000	400	400	Berendsen	Berendsen
5	50	1000	1000	400	200	Berendsen	Berendsen
6	300	500	500	200	200	Berendsen	Berendsen
7	300	200	200	40	100	Berendsen	Berendsen
8	300	200	200	0	0	Berendsen	Berendsen
9	10000	200	50	0	0	Berendsen	Berendsen
10	20000	50	0	0	0	Berendsen	Berendsen

a All restraints are harmonic. Distance force constants have units of kJ/(mol·nm^2^), and dihedral force constants are in units of kJ/(mol rad^2^). *Residue PC atoms [C1, C2, C3, O21], residue PA atoms [C114, H16S, H15R, H14S].

#### Production

Production simulations used the stochastic v-rescale thermostat^26^ and Parrinello–Rahman barostat.^27^

#### Weighted Ensemble

Each equilibrated system was used as the starting structure for two weighted ensemble simulations run using WESTPA 2.0.^11^ The distance from the ligand to its binding site in the energy-minimized structure, measured by the ligand’s center of mass, was used as the progress coordinate. As the center of mass included the R group, the arbitrary initial tail orientation may have introduced small artifacts into the progress coordinate values of CFTRi-C10. Fixed bins as described in Table 2 were used with a target of 4 walkers per bin and a WESTPA round length of 50 ps. Simulations were periodically reweighted using WESTPA’s Weighted Ensemble Equilibrium Dynamics module.^28^

### Data Processing

Scripts used to numerically analyze the kinetic model of ABV-974 dissociation, as well as MD simulation input files and analysis scripts, are at https://github.com/JonathanHB/cftr-potentiator-SAR.

## Supplementary Material











## Notes

Additional MD simulation analysis, experimental data, synthetic procedures, and characterization data (PDF)
(Video S1) MD simulation trajectories (MOV)
(Video S2) MD simulation trajectories (MOV)
(Video S3) MD simulation trajectories (MOV)
(Video S4) MD simulation trajectories (MOV)

## References

[1] Santos R., Ursu O., Gaulton A., Bento A. P., Donadi R. S., Bologa C. G., Karlsson A., Al-Lazikani B., Hersey A., Oprea T. I., Overington J. P. (2017). A Comprehensive
Map of Molecular Drug Targets. Nat. Rev. Drug
Discovery.

[2] Payandeh J., Volgraf M. (2021). Ligand Binding at the Protein–Lipid Interface:
Strategic Considerations for Drug Design. Nat.
Rev. Drug Discovery.

[3] Villemure E., Terrett J. A., Larouche-Gauthier R., Déry M., Chen H., Reese R. M., Shields S. D., Chen J., Magnuson S., Volgraf M. (2021). A Retrospective Look
at the Impact
of Binding Site Environment on the Optimization of TRPA1 Antagonists. ACS Med. Chem. Lett..

[4] Szlenk C. T., Gc J. B., Natesan S. (2019). Does the Lipid
Bilayer Orchestrate
Access and Binding of Ligands to Transmembrane Orthosteric/Allosteric
Sites of G Protein-Coupled Receptors?. Mol.
Pharmacol..

[5] Hadida S., Van Goor F., Zhou J., Arumugam V., McCartney J., Hazlewood A., Decker C., Negulescu P., Grootenhuis P. D. J. (2014). Discovery of N-(2,4-Di-Tert-Butyl-5-Hydroxyphenyl)-4-Oxo-1,4-Dihydroquinoline-3-Carboxamide
(VX-770, Ivacaftor), a Potent and Orally Bioavailable CFTR Potentiator. J. Med. Chem..

[6] Van
der Plas S. E., Kelgtermans H., De Munck T., Martina S. L. X., Dropsit S., Quinton E., De Blieck A., Joannesse C., Tomaskovic L., Jans M., Christophe T., van der Aar E., Borgonovi M., Nelles L., Gees M., Stouten P., Van Der Schueren J., Mammoliti O., Conrath K., Andrews M. (2018). Discovery of N-(3-Carbamoyl-5,5,7,7-Tetramethyl-5,7-Dihydro-4H-Thieno­[2,3-c]­Pyran-2-Yl)-lH-Pyrazole-5-Carboxamide
(GLPG1837), a Novel Potentiator Which Can Open Class III Mutant Cystic
Fibrosis Transmembrane Conductance Regulator (CFTR) Channels to a
High Extent. J. Med. Chem..

[7] Liu F., Zhang Z., Levit A., Levring J., Touhara K. K., Shoichet B. K., Chen J. (2019). Structural
Identification of a Hotspot
on CFTR for Potentiation. Science.

[8] Liu F., Kaplan A. L., Levring J., Einsiedel J., Tiedt S., Distler K., Omattage N. S., Kondratov I. S., Moroz Y. S., Pietz H. L., Irwin J. J., Gmeiner P., Shoichet B. K., Chen J. (2024). Structure-Based
Discovery of CFTR
Potentiators and Inhibitors. Cell.

[9] Yeh H.-I., Sohma Y., Conrath K., Hwang T.-C. (2017). A Common Mechanism
for CFTR Potentiators. J. Gen. Physiol..

[10] Yeh H.-I., Qiu L., Sohma Y., Conrath K., Zou X., Hwang T.-C. (2019). Identifying
the Molecular Target Sites for CFTR Potentiators GLPG1837 and VX-770. J. Gen. Physiol..

[11] Russo J. D., Zhang S., Leung J. M. G., Bogetti A. T., Thompson J. P., DeGrave A. J., Torrillo P. A., Pratt A. J., Wong K. F., Xia J., Copperman J., Adelman J. L., Zwier M. C., LeBard D. N., Zuckerman D. M., Chong L. T. (2022). WESTPA 2.0: High-Performance Upgrades
for Weighted Ensemble Simulations and Analysis of Longer-Timescale
Applications. J. Chem. Theory Comput.

[12] Kim S., Chen J., Cheng T., Gindulyte A., He J., He S., Li Q., Shoemaker B. A., Thiessen P. A., Yu B., Zaslavsky L., Zhang J., Bolton E. E. (2023). PubChem 2023 update. Nucleic Acids Res..

[13] Morstein J., Capecchi A., Hinnah K., Park B., Petit-Jacques J., Van Lehn R. C., Reymond J.-L., Trauner D. (2022). Medium-Chain Lipid
Conjugation Facilitates Cell-Permeability and Bioactivity. J. Am. Chem. Soc..

[14] Morstein J., Shrestha R., Van Q. N., López C. A., Arora N., Tonelli M., Liang H., Chen D., Zhou Y., Hancock J. F., Stephen A. G., Turbyville T. J., Shokat K. M. (2023). Direct Modulators of K-Ras–Membrane
Interactions. ACS Chem. Biol..

[15] DeLano W. L., Lam J. W. (2005). PyMOL: A Communications Tool for Computational Models. Abstr. Pap. Am. Chem. Soc..

[16] Lee J., Cheng X., Swails J. M., Yeom M. S., Eastman P. K., Lemkul J. A., Wei S., Buckner J., Jeong J. C., Qi Y., Jo S., Pande V. S., Case D. A., Brooks C. L., MacKerell A. D., Klauda J. B., Im W. (2016). CHARMM-GUI Input Generator for NAMD, GROMACS, AMBER, OpenMM, and
CHARMM/OpenMM Simulations Using the CHARMM36 Additive Force Field. J. Chem. Theory Comput.

[17] Lee J., Hitzenberger M., Rieger M., Kern N. R., Zacharias M., Im W. (2020). CHARMM-GUI Supports the Amber Force Fields. J. Chem. Phys..

[18] Dickson C. J., Walker R. C., Gould I. R. (2022). Lipid21: Complex Lipid Membrane Simulations
with AMBER. J. Chem. Theory Comput.

[19] Wang J., Wolf R. M., Caldwell J. W., Kollman P. A., Case D. A. (2004). Development
and Testing of a General Amber Force Field. J. Comput. Chem..

[20] Jorgensen W. L., Chandrasekhar J., Madura J. D., Impey R. W., Klein M. L. (1983). Comparison
of Simple Potential Functions for Simulating Liquid Water. J. Chem. Phys..

[21] Joung I. S., Cheatham T. E. (2008). Determination of Alkali and Halide
Monovalent Ion Parameters for Use in Explicitly Solvated Biomolecular
Simulations. J. Phys. Chem. B.

[22] Abraham M.
J., Murtola T., Schulz R., Páll S., Smith J. C., Hess B., Lindahl E. (2015). GROMACS: High Performance
Molecular Simulations through Multi-Level Parallelism from Laptops
to Supercomputers. SoftwareX.

[23] Hess B., Bekker H., Berendsen H. J. C., Fraaije J. G. E. M. (1997). LINCS: A Linear
Constraint Solver for Molecular Simulations. J. Comput. Chem..

[24] Darden T., York D., Pedersen L. (1993). Particle Mesh
Ewald - an N.Log­(N)
Method for Ewald Sums in Large Systems. J. Chem.
Phys..

[25] Berendsen H. J. C., Postma J. P. M., van Gunsteren W. F., DiNola A., Haak J. R. (1984). Molecular
Dynamics with Coupling to an External Bath. J. Chem. Phys..

[26] Bussi G., Donadio D., Parrinello M. (2007). Canonical
Sampling through Velocity
Rescaling. J. Chem. Phys..

[27] Parrinello M., Rahman A. (1981). Polymorphic Transitions
in Single Crystals: A New Molecular
Dynamics Method. J. Appl. Phys..

[28] Bhatt D., Zhang B. W., Zuckerman D. M. (2010). Steady-State
Simulations Using Weighted
Ensemble Path Sampling. J. Chem. Phys..

