# Supplementary material for: Targeting the Protein–Membrane Interface Enables Design of Long-Acting CFTR Potentiators
Source: ACS Chem Biol. 2026 Apr 28;21(5):982–9. doi: 10.1021/acschembio.5c00993 (PMC13184935; doi:10.1021/acschembio.5c00993)
Supplement: Supplementary file 1 [file cb5c00993_si_001.pdf]

Supporting Information

Targeting the Protein-Membrane Interface Enables Design of Long-Acting CFTR Potentiators

Johannes Morstein[a,b*,#], Jonathan Borowsky[c,#], Shenghui Hu[d,#], YooJin Sheen[c] ,

Victoria Nisoli[b], Tzyh-Chang Hwang[d,*], Michael Grabe[c,*], Kevan M. Shokat[a,*]

[a] Department of Cellular and Molecular Pharmacology and Howard Hughes Medical Institute, University of California, San Francisco, California 94158, USA

[b] Division of Chemistry and Chemical Engineering, California Institute of Technology, Pasadena, California 91125, USA;

[c] Department of Pharmaceutical Chemistry and Cardiovascular Research Institute, University of California, San Francisco, California 94143, United States

[d] Dalton Cardiovascular Research Center, University of Missouri-Columbia, Columbia, MO, 65211, USA

#These authors contributed equally

*morstein@caltech.edu; thwang@health.missouri.edu; Michael.Grabe@ucsf.edu;

Kevan.Shokat@ucsf.edu

A mathematical model relating electrophysiological CFTR current decay time constants to octanol-water partitioning coefficients of potentiator R groups

Section 1: Relation of partitioning coefficients to partitioning rates for pairs of chemical analogues

Section 1.1: Partitioning coefficients (LogP). For solutions of water (w) and octanol (o), containing dissolved ligand L, let $[L_w]$ be the ligand concentration in water and $[L_o]$ be its concentration in octanol. Then the ratio is:

$$\mathrm{P} \equiv \frac{[\mathrm{L_o}]}{[\mathrm{L_w}]}$$

$$logP \equiv log_{10}(P) \quad eq. 1$$

The Gibbs free energy (ΔG_{wo}) of partitioning from water to octanol is:

$$e^{-\Delta G_{wo}/k_B T} \equiv P \quad e\mathrm{q}. 2$$

where T is temperature, and k_B is the Boltzmann constant. The ratio of partitioning coefficients of two molecules, denoted R1 and R2, is then:

$$\frac{P_{R1}}{P_{R2}} = \frac{e^{-\Delta G_{woR1}/k_B T}}{e^{-\Delta G_{woR2}/k_B T}} = e^{-(\Delta G_{woR1} - \Delta G_{woR2})/k_B T} \quad eq. 3$$

Section 1.2: Rates. Consider a molecule which can transition between solvent phases i and j. The rate constant for the transition can be expressed using a prefactor r and activation free energy $\Delta G^{\ddagger}_{\mathrm{ij}}$:

$$\mathrm{k_{ij}} = r\mathrm{e}^{-\Delta \mathrm{G}^{\ddagger}_{ij}/\mathrm{k_B T}} \quad eq. 4$$

In the absence of knowledge of the microscopic details of the partitioning process, it is reasonable to assume that the equilibrium free energy difference is split equally between the activation energies of the forward and reverse rates:

$$\Delta G^{\ddagger} + \Delta G_{ij}/2 \equiv \Delta G^{\ddagger}_{ij} \quad \text{and} \quad \Delta G^{\ddagger} - \Delta G_{ij}/2 \equiv \Delta G^{\ddagger}_{ji} \quad eq. 5\, a \text{ and } b$$

Section 1.3: Ratios of membrane to water partitioning rates. Consider a small molecule which can transition between a lipid bilayer membrane and water (or other aqueous solution like cytoplasm or extracellular fluid), denoted l and w. Using equation 4 (where i=l and j=w) the membrane to water transition rate constant is:

$$\mathrm{k_{lw}} = r\mathrm{e}^{-\Delta \mathrm{G_{lw}}^{\ddagger}/\mathrm{k_B T}} \quad eq.6$$

From equation 5b:

$$\Delta G^{\ddagger}_{lw} = \Delta G^{\ddagger} - \Delta G_{wl}/2 \quad eq.7$$

Consider two molecules, denoted 1 and 2, composed of the same bulky chemical group M and a variable R group which distinguishes them. Suppose that the free energy contributions of the two groups are roughly additive such that the free energy of partitioning of each molecule can be broken into M- and R-group dependent terms:

$$\Delta G_{wl} = \Delta G_{wlM} + \Delta G_{wlR} \quad eq.8$$

$\Delta G^{\ddagger}$ is the free energy cost of a molecule being in the transition state between water and the membrane. This is the cost of occupying the transition state specifically after accounting for the additive free energy of being partially in water and partially in the membrane. Assume that the hydrophobic effect is roughly additive as a molecule moves along the membrane normal because the molecule can progressively form or break hydrogen bonds (and decrease the entropy of the water hydrogen bonding network) in proportion to how much of it sticks out of the membrane. Further suppose that partitioning of small R-group-sized molecules between water and phospholipid membranes is barrierless because they can fit between the esters and initial carbons of the fatty acid tails which form the most tightly packed region of the membrane with minimal displacement. The transition state free energy $\Delta G^{\ddagger}$ is then mainly a reflection of the energetic cost of forming a membrane packing defect large enough to admit the bulky M group (or other M group specific interactions), making it R group independent (specifically $\Delta G^{\ddagger}_{R1},\ \Delta G^{\ddagger}_{R2} \ll k_B T$):

$$\Delta G^{\ddagger}_{1} = \Delta G^{\ddagger}_{M} = \Delta G^{\ddagger}_{2} \quad eq.9$$

Consider the ratio of membrane-to-water partitioning rates of these two molecules, using equation 6, substitute in equations 7, 8, and 9, and cancel the prefactors (which are assumed to reflect the diffusion coefficient of the bulky M group and self-diffusion coefficients of water and lipids) and other R group independent terms:

$$\frac{k_{lw1}}{k_{lw2}} = \mathrm{e}^{-(\Delta \mathrm{G_{lw1}}^{\ddagger} - \Delta \mathrm{G_{lw2}}^{\ddagger})/\mathrm{k_B T}} = \mathrm{e}^{-(-\Delta G_{wlR1} + \Delta G_{wlR2})/2\mathrm{k_B T}} \quad eq.10$$

Section 1.4: Membrane to water partitioning rate ratios in terms of logP. Suppose that logP is an accurate proxy for partitioning between water and the membrane such

that $\Delta G_{wlR} = \Delta G_{woR}$ up to some R group independent term reflecting the fact that bulk octanol is three dimensional and the membrane is two dimensional. Equations 3 and 10 are then related as follows:

$$\frac{k_{lw1}}{k_{lw2}} = \mathrm{e}^{-(-\Delta G_{woR1}+\Delta G_{woR2})/2\mathrm{k_B T}} = \left(\frac{P_{R2}}{P_{R1}}\right)^{1/2} \quad eq. 11.1$$

Converting from P to logP using eq. 1:

$$\boldsymbol{\frac{k_{lw1}}{k_{lw2}} = \frac{10^{logP_{2R}/2}}{10^{logP_{1R}/2}} \quad eq\ 11.2}$$

Section 2: Kinetic model of CFTR ligand binding

Section 2.1: Definitions.

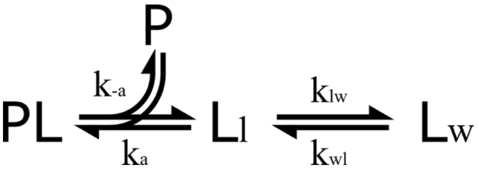

P = protein

L = ligand

PL = protein-ligand complex

L_l = ligand in membrane

L_w = ligand in water

Figure A1: Diagram of the states of the protein and ligand and the rate constants of the transitions between them.

CFTR channels were initially incubated in a saturating concentration of ligand L_w allowing the system (protein, membrane, aqueous states) to reach equilibrium, and then channel activity was recorded via patch clamp electrophysiology as ligand was continually washed out/removed from the aqueous solution. A single exponential was fit to the current trace from the period of ligand removal, yielding a time constant T. Instances of the variable 'T' below refer to this time constant rather than the temperature. In washout experiments, we assume that the aqueous ligand concentration $[L_w]$ is zero, and the dynamics of the system are then described by the following differential equations:

$$[\dot{PL}] = -k_{-a}[PL] + k_a[L_l][P] \quad eq. 12a$$

$$[\dot{L_l}] = k_{-a}[PL] - k_a[L_l][P] - k_{lw}[L_l] \quad eq. 12b$$

where dots denote time derivatives. Substituting eq. 12b into eq. 12a to eliminate $[L_l]$ yields:

$$\left([\dot{PL}] + [\dot{L_l}]\right)\mathrm{k_a}[\mathrm{P}] + [\dot{PL}]\mathrm{k_{lw}} = -k_{lw}k_{-a}[PL] \quad eq. 13$$

The total amount of protein is a constant as it remains embedded in the membrane:

$$[P_t] \equiv [P] + [PL] \quad eq. 14$$

Suppose that the experimental current (or the ligand-dependent portion of the current to which the exponential was fit) is proportional to the ligand-bound protein concentration [PL], and is normalized by the maximal current:

$$x \equiv \frac{[PL]}{[P_t]} \quad eq. 15$$

<u>Section 2.2: Quasi steady state dynamics.</u> Suppose that the potentiator affinity for the protein is high such that $k_a[P_t] >> k_{-a}$. Then during washout experiments, once the initial excess of ligand has left the system and apo protein concentration [P] is non-zero, $k_a[P] >> k_{-a}$. In a washout experiment, this means that, after initial transients, [PL] is always much greater than $[L_l]$ due to the ligand's favorable binding to the protein. As both [PL] and $[L_l]$ decrease smoothly and monotonically to 0 as all ligand washes out, it follows that $[\dot{PL}] \gg [\dot{L_l}]$. Applying this result to eq. 13. yields:

$$[\dot{PL}] = \frac{-k_{-a}k_{lw}[PL]}{k_{lw} + k_a[P]} \quad eq. 16$$

Substituting eqs. 14 and 15 into eq. 16 to change variables yields:

$$\dot{x} = \frac{-k_{-a}k_{lw}x}{k_{lw} + k_a[P_t](1-x)} \quad eq. 17a$$

This equation can be integrated as follows, but there exists no closed form analytic solution in terms of elementary functions for x in terms of t:

$$-k_{-a}k_{lw}t = (k_{lw} + k_a[P_t])ln\left(\frac{x}{x_0}\right) - k_a[P_t](x - x_0) \quad eq. 17b$$

The behavior of the system is determined by the relative values of k_{lw} and $k_a[P_t]$.

Section 2.3: Fast washout limit. **In the limit of $k_{lw} \gg k_a[P_t]$** , eq. 17a reduces to:

$$\dot{x} = \frac{-k_{-a}k_{lw}x}{k_{lw}} = -k_{-a}x$$

which has the solution:

$$x = x_0 e^{-k_{-a}t}$$

In this limit, ligands that dissociate from the protein immediately leave the membrane and wash out. The current decay is then a single exponential with time constant $T = 1/\mathrm{k}_{-\mathrm{a}}$.

Section 2.4.1: Fast rebinding limit. **In the limit of $k_{lw} \ll k_a[P_t]$** , equation 17a reduces to:

$$dt = -\frac{k_a[P_t]}{k_{-a}k_{lw}}\frac{1-x}{x}dx \quad eq. 18a$$

And equation 17b reduces to:

$$-k_{-a}k_{lw}t = k_a[P_t]ln\left(\frac{x}{x_0}\right) - k_a[P_t](x - x_0) \quad eq. 18b$$

For a protein-ligand dissociation constant $K_d \equiv \frac{k_{-a}}{k_a}$, this equals:

$$-K_d\frac{k_{lw}}{[P_t]}\mathrm{t} = \ln\left(\frac{x}{x_0}\right) - (x - x_0)$$

This implies that as long as $k_a[P_t]$ remains much larger than k_{lw}, the washout rate depends on the thermodynamics of the ligand binding to the protein from the membrane but not on the absolute kinetics of this process.

As $x \to 0$, current decay is modeled exactly by a single exponential for which the time constant is proportional to $1/k_{lw}$:

$$-\frac{k_{-a}k_{lw}}{k_a[P_t]}\mathrm{t} - x_0 = \ln\left(\frac{x}{x_0}\right); \quad x = x_0 e^{-\left(\frac{k_{-a}k_{lw}}{k_a[P_t]}t + x_0\right)} = x_0 e^{-x_0} e^{-\frac{k_{-a}k_{lw}}{k_a[P_t]}t}$$

However, collecting data solely in the limit of $x \to 0$ is impractical since the signal and hence the signal-to-noise ratio go to 0.

Section 2.4.2: Fast rebinding limit: Least squares exponential fit. While equation 18b is not exponential, numerical analysis indicates that exponential curves provide good approximations to it. If an exponential curve $\hat{x} = Ae^{-t/T}$ is fit to it, the mean squared error will be:

$$\mathrm{MSE} = \int_0^\infty (\hat{x} - x)^2 dt \ = \int_0^\infty \left(\mathrm{Ae}^{-\mathrm{t/T}} - x\right)^2 dt \quad eq. 19$$

For direct least squares regression (*not* via linear regression to a linearized equation), MSE will be minimized with respect to A and T, so its derivatives with respect to them will be 0.

Derivative with respect to T:

$$\frac{\mathrm{dMSE}}{\mathrm{dT}} = \int_0^\infty \frac{d}{dT}(\hat{x} - x)^2 dt \ = 2\int_0^\infty \frac{d\hat{x}}{dT}(\hat{x} - x) dt$$

$$= 2\int_0^\infty \frac{\mathrm{At}}{\mathrm{T}^2}\mathrm{e}^{-\mathrm{t/T}}\left(A\mathrm{e}^{-\mathrm{t/T}} - \mathrm{x}\right)\mathrm{dt} \quad eq. 20a$$

Let $\alpha \equiv \frac{k_a[P_t]}{k_{-a}k_{lw}}$ and $f(x, x_0) \equiv ln\left(\frac{x}{x_0}\right) - (x - x_0)$

Substituting α and $f(x, x_0)$ into eq. 18a and 18b:

$$dt = -\alpha\frac{1 - x}{x}dx$$

$$t = -\alpha f(x, x_0)$$

Substituting the above into eq. 20a to change variables:

$$\frac{\mathrm{dMSE}}{\mathrm{dT}} = 2\int_1^0 \frac{\mathrm{A\alpha f(x, x_0)}}{\mathrm{T}^2}\mathrm{e}^{\alpha\mathrm{f(x,x_0)/T}}\left(\mathrm{Ae}^{\alpha\mathrm{f(x,x_0)/T}} - \mathrm{x}\right)\alpha\frac{1 - \mathrm{x}}{\mathrm{x}}\mathrm{dx}$$

$$= 2\mathrm{A}\int_{\mathrm{x}_0}^0 \left(\frac{\alpha}{\mathrm{T}}\right)^2 \mathrm{e}^{\mathrm{f(x,x_0)\alpha/T}}\left(\mathrm{Ae}^{\mathrm{f(x,x_0)\alpha/T}} - \mathrm{x}\right)\mathrm{f(x, x_0)}\frac{1 - \mathrm{x}}{\mathrm{x}}\mathrm{dx}$$

Let $\beta \equiv \alpha / T$:

$$\frac{\mathrm{dMSE}}{\mathrm{dT}} \ = \ 2\mathrm{A}\int_{\mathrm{x}_0}^0 \beta^2\mathrm{e}^{\beta\,\mathrm{f(x,x_0)}}\left(\mathrm{Ae}^{\beta\,\mathrm{f(x,x_0)}} - \mathrm{x}\right)\mathrm{f(x, x_0)}\frac{1 - \mathrm{x}}{\mathrm{x}}\mathrm{dx}$$

Minimizing MSE with respect to T:

$$0 = \int_{\mathrm{x}_0}^0 \mathrm{e}^{\beta\,\mathrm{f(x,x_0)}}\left(\mathrm{Ae}^{\beta\,\mathrm{f(x,x_0)}} - \mathrm{x}\right)\mathrm{f(x, x_0)}\frac{1 - \mathrm{x}}{\mathrm{x}}\mathrm{dx} \quad eq. 20b$$

Derivative with respect to A:

$$\frac{\mathrm{dMSE}}{\mathrm{dA}} = \int_0^\infty \frac{d}{dA}(\hat{x} - x)^2 dt \ = 2\int_0^\infty \frac{d\hat{x}}{dA}(\hat{x} - x)dt = 2\int_0^\infty \mathrm{e}^{-\mathrm{t/T}}\left(\mathrm{Ae}^{-\mathrm{t/T}} - \mathrm{x}\right)\mathrm{dt} \quad eq. 21a$$

Substituting as above and minimizing MSE with respect to A:

$$0 = \int_{x_0}^{0} e^{\beta\, f(x,x_0)} \left(Ae^{\beta\, f(x,x_0)} - x\right) \frac{1-x}{x} dx \qquad eq. 21b$$

eq. 20b and eq. 21b have some solution (A_{mse}, β_{mse}) for a given x_0. For a saturating initial ligand concentration, x_0 = 1. Let the solution then be denoted (A_{mse1}, β_{mse1}). This solution is determined only the relative shapes of the exponential curve and the functional form of eq. 18b and is thus independent of all the individual biological parameters comprising β. The relationship between k_{lw} and T for any experiment run at a saturating ligand concentration is thus:

$$\beta_{mse1} = \frac{k_a[P_t]}{k_{-a}k_{lw}T} = \frac{[P_t]}{K_d k_{lw}T}; \quad \boldsymbol{k_{lw}} = \frac{[\boldsymbol{P_t}]}{\boldsymbol{K_d \beta_{mse1} T}} \qquad \boldsymbol{eq. 22}$$

Numerical analysis indicates that $\beta_{mse1} \cong 1.53$, but this factor cancels in the final analysis presented in the next subsection.

Section 2.4.3: Fast rebinding limit: Ratios of time constants for ligand analogues.

Substituting eq. 22 into eq. 11.2 we have:

$$\frac{[P_t]_1/(K_{d1}\beta_{mse1}T_1)}{[P_t]_2/(K_{d2}\beta_{mse1}T_2)} = \frac{[P_t]_1/(K_{d1}T_1)}{[P_t]_2/(K_{d2}T_2)} = \frac{10^{logP_{2R}/2}}{10^{logP_{1R}/2}} \quad eq. 23.1$$

Suppose that K_d is R-group independent because the hydrophobic R groups are equally at home on the hydrophobic protein surface and in the membrane. In the case of CFTR, this assumption is also supported by the fact that the binding site is relatively flat and lacks deep pockets or grooves which would be expected to have large R-group-specific effects on binding free energy. If the experiments are also carried out at equal total protein concentrations $[P_t]$, then eq. 23.1 reduces to:

$$\boldsymbol{\frac{T_2}{T_1} = \frac{10^{logP_{2R}/2}}{10^{logP_{1R}/2}} \quad eq. 23.2}$$

This ratio equality implies that for a series of analogues i and some constant m:

$$T_i = 10^{logP_{iR}/2}\mathrm{m} \quad \mathrm{eq.} 23.3$$

Thus, if T_i is plotted against $10^{logP_{iR}/2}$, all analogues will lie on a line of slope m and intercept (0,0). The value of m depends on absolute rates and other parameters which cancelled out above and are usually not known. To estimate m for multiple analogues while weighting the data for all analogues equally, calculate m for each analogue and take the average. The use of linear regression (with a regression equation $\hat{y} = mx$) to estimate m is inadvisable because this will give more weight to analogues with larger absolute T values. Also note that the intercept (0,0) is unphysical both because it represents the limit of infinitely hydrophilic ligands and because the fast rebinding limit would presumably eventually cease to hold as the ligands became increasingly hydrophilic.

Molecular dynamics simulation analysis

Figure S1. Protein contacts and water penetration as a function of ligand distance from bound pose. Data for ABBV-974 run 1 (A), run 2 (B), CFTRi-C10 run 1 (C), and run 2 (D). Contacts are calculated between heavy atoms using a 5 Å cutoff distance. Contact frequencies (these are dimensionless probabilities of being in contact, not a dynamical quantity with units of inverse time) were obtained by averaging binary contact maps (with equal weights; as the Weighted Ensemble (WE) weights are not converged) across all frames (subsampling to every 10th WE round) in each weighted ensemble progress coordinate (PC) range. White represents a contact frequency of zero, while fully saturated blue, green, and red represent contact frequencies of one (i.e. the protein is in contact with an atom from the specified type of molecule in all frames). Frames in the rightmost column are taken from the reactive trajectory leading to the farthest-dissociated walker (selecting the chronological median from among all reactive trajectory frames in the PC range). R933 and E873 are shown as sticks in the rightmost column (and are the only Arg/Glu residues shown).

Figure S1A. Contacts and frames from ABBV-974 run 1.

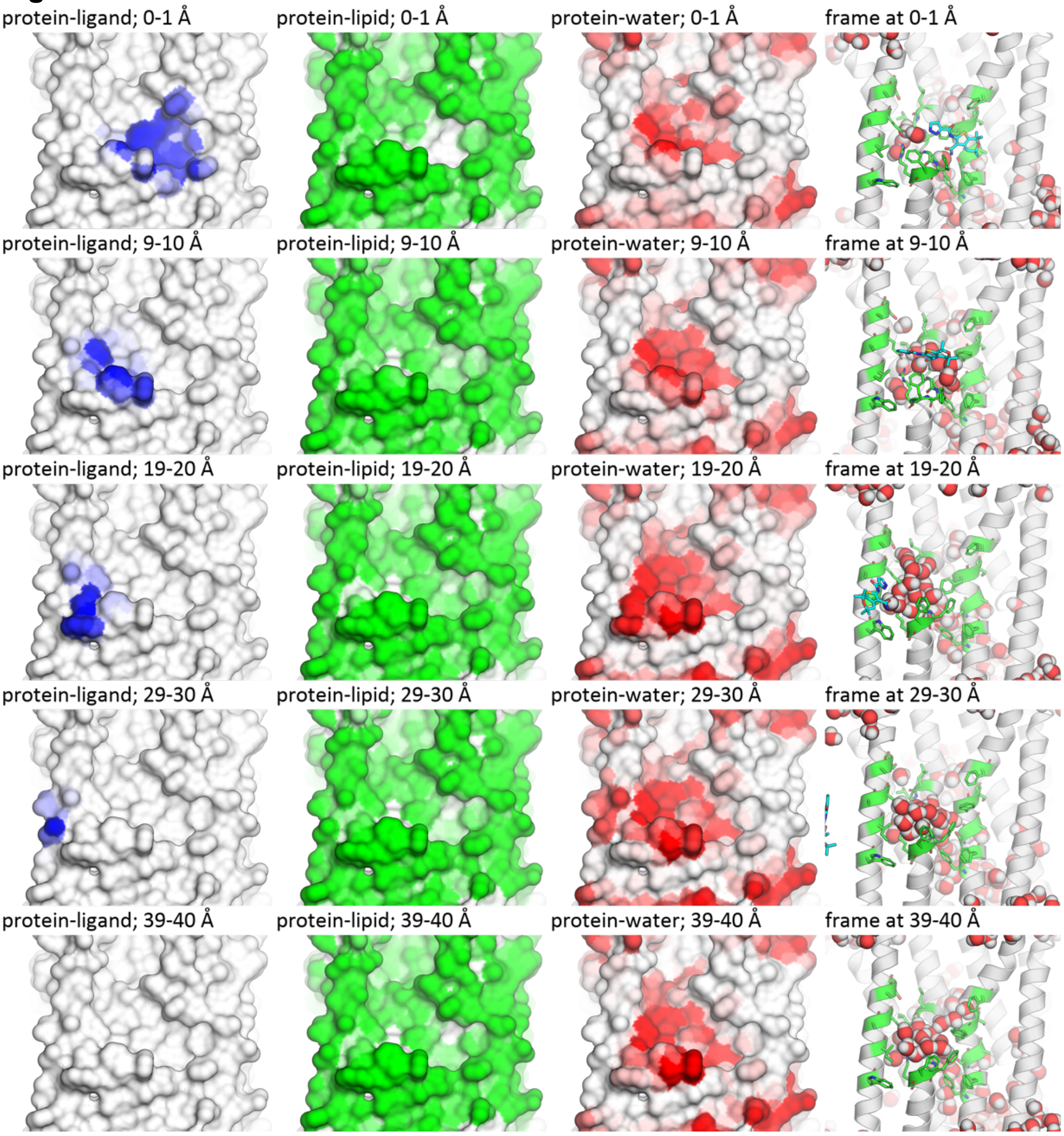

Figure S1B. Contacts and frames from ABBV-974 run 2.

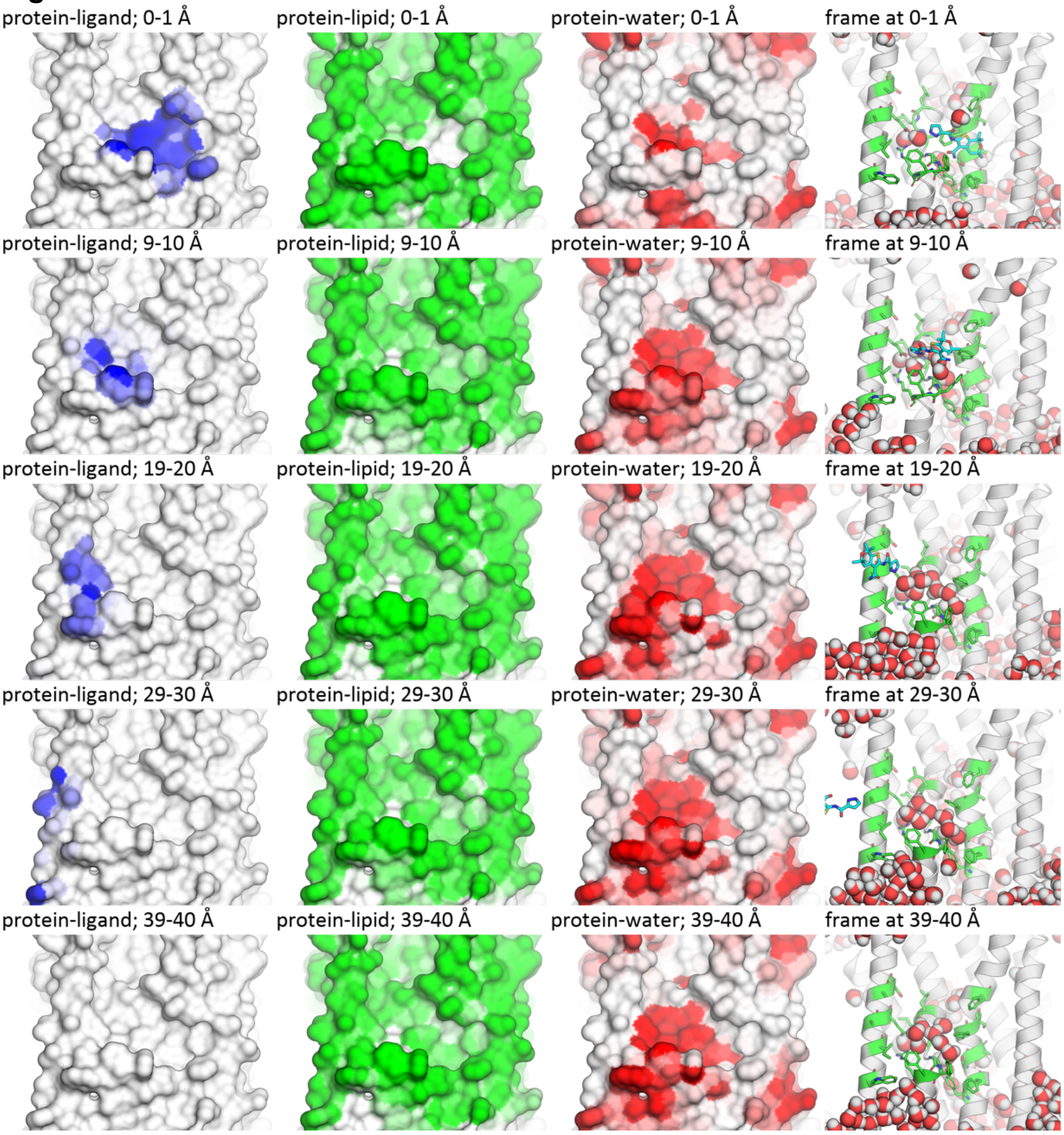

Figure S1C. Contacts and frames from CFTRi-C10 run 1.

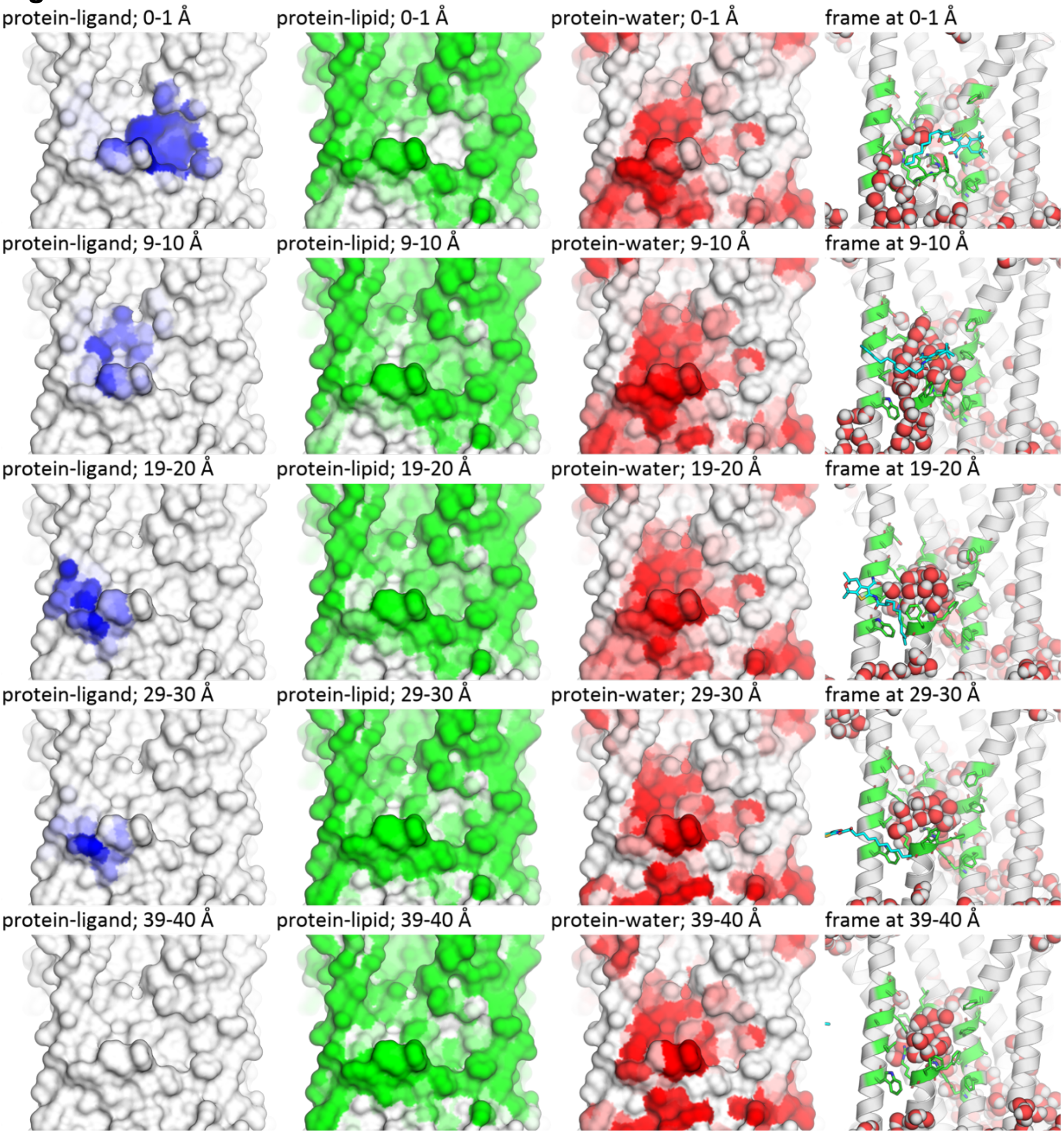

Figure S1D. Contacts and frames from CFTRi-C10 run 2.

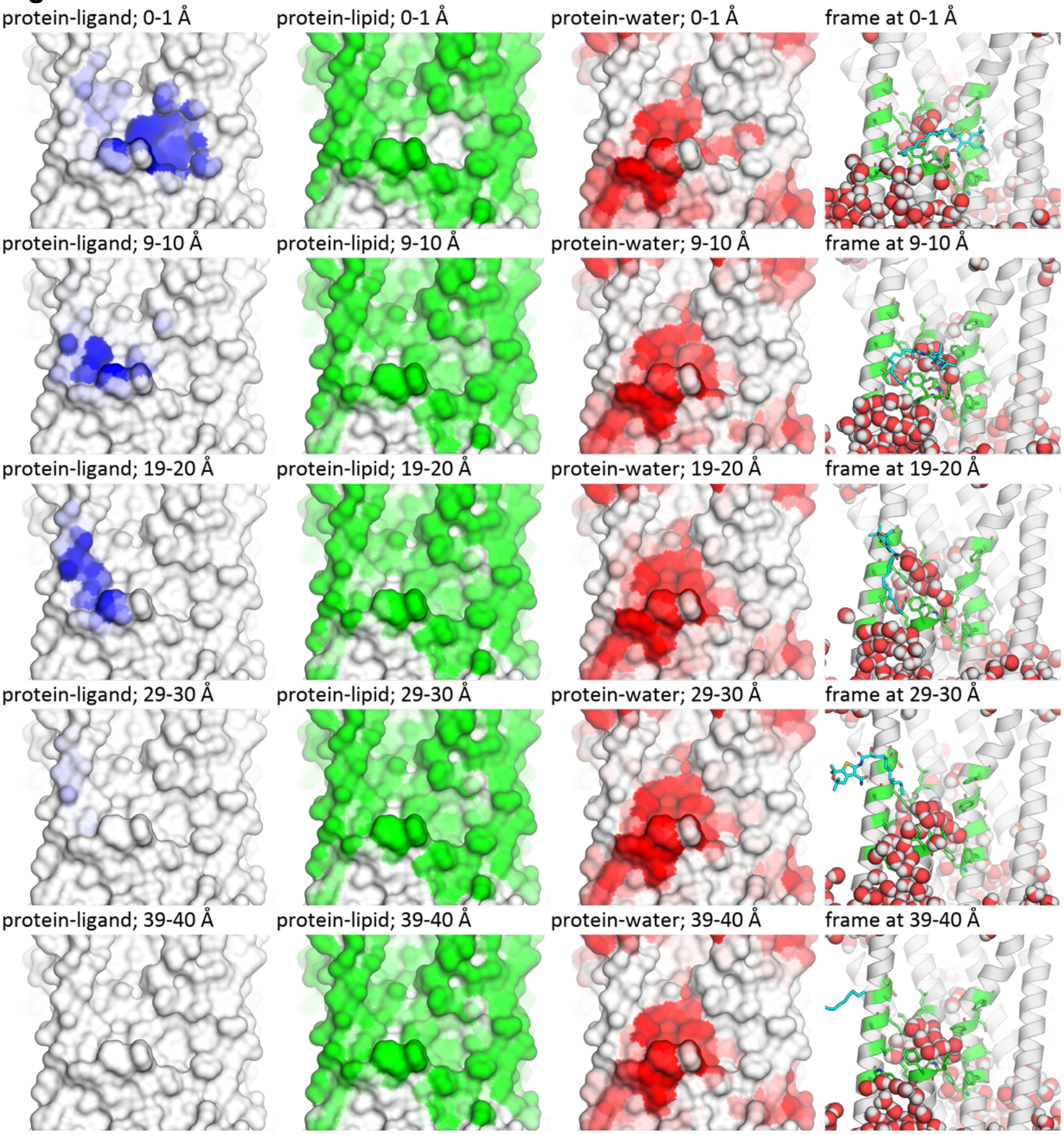

Figure S2. Membrane and box dimensions as a function of progress coordinate (PC). All frames from every 10th WE round were included, and all frames in each PC range were weighted equally. Error bars are ±1 SD. Y axes are shared across rows, and x axes are shared across columns. Note that the plots in the upper and lower rows are strongly negatively correlated because changing the membrane volume is more energetically costly than changing its aspect ratio.

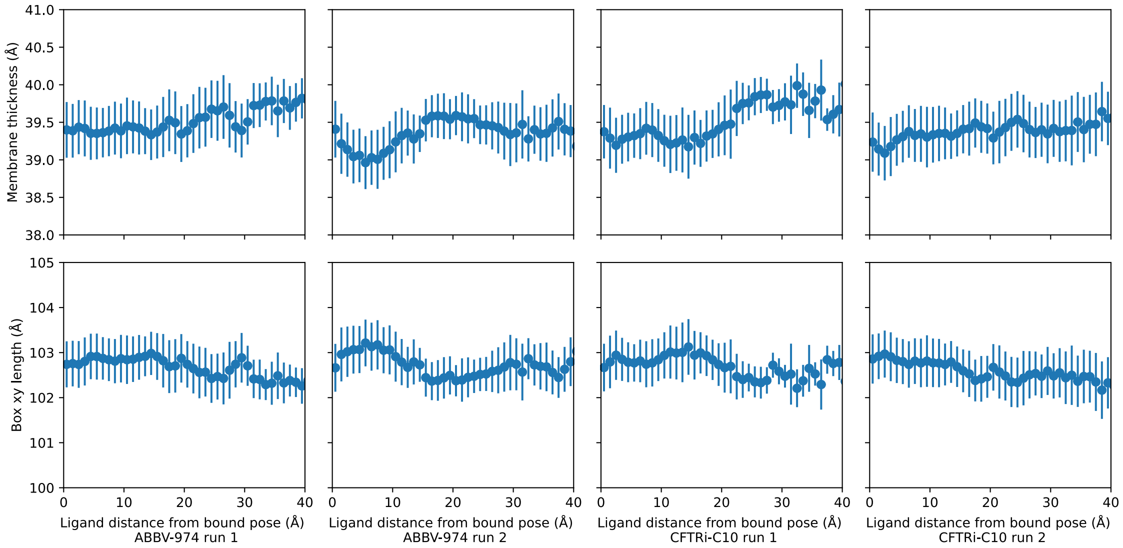

Figure S3. Hydrogen bond probability between R group polar atoms and E873 or R933. Hydrogen bonds are defined as heavy atom distances < 3.3 Å between donor-acceptor-capable atoms. Probabilities were calculated by averaging binary hydrogen bond states across frames. All frames from every 10th Weighted Ensemble (WE) round were included, with equal weighting within each PC range. Error bars indicate ±1 SD. Y axes are shared across rows, and x axes are shared across columns.

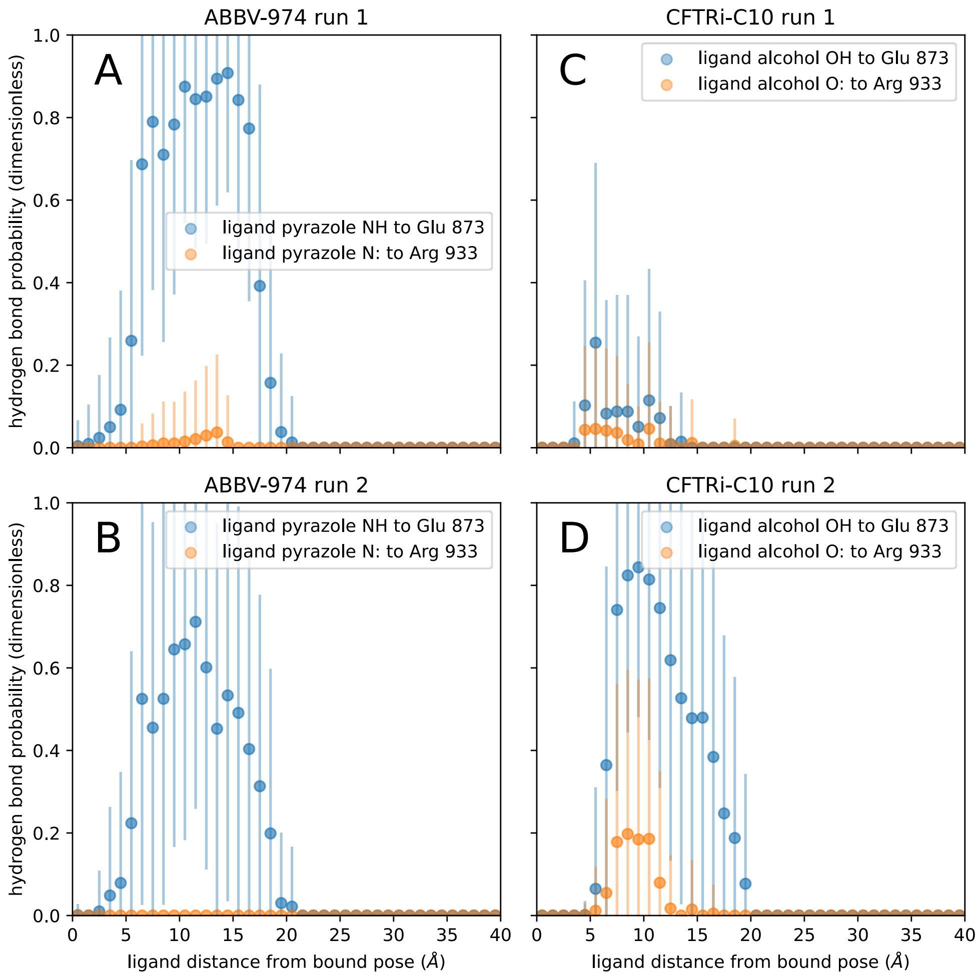

Figure S4. Ligand protein, lipid FA, and water contacts. Contact frequencies are calculated and rendered as in Fig. S1.

Figure S4A. Contacts from ABBV-974 run 1.

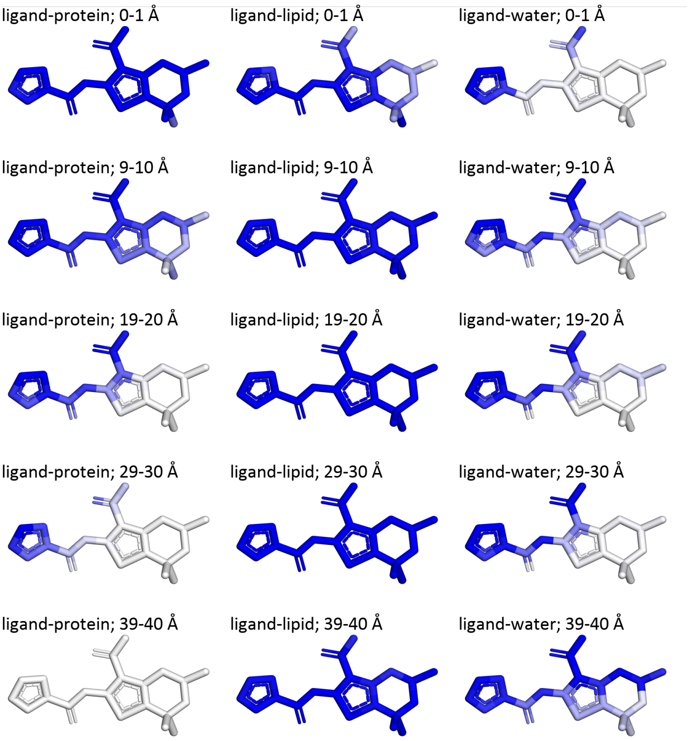

Figure S4B. Contacts from ABBV-974 run 2.

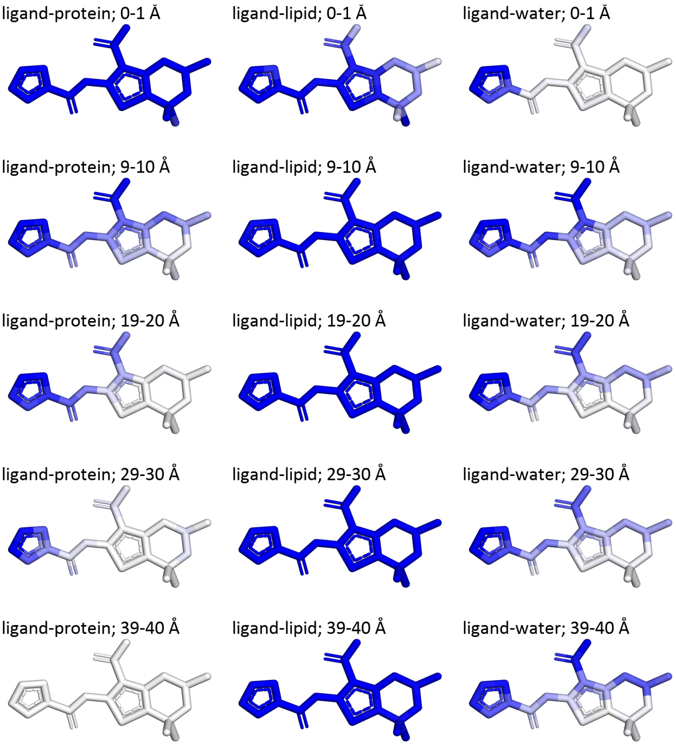

Figure S4C. Contacts from CFTRi-C10 run 1.

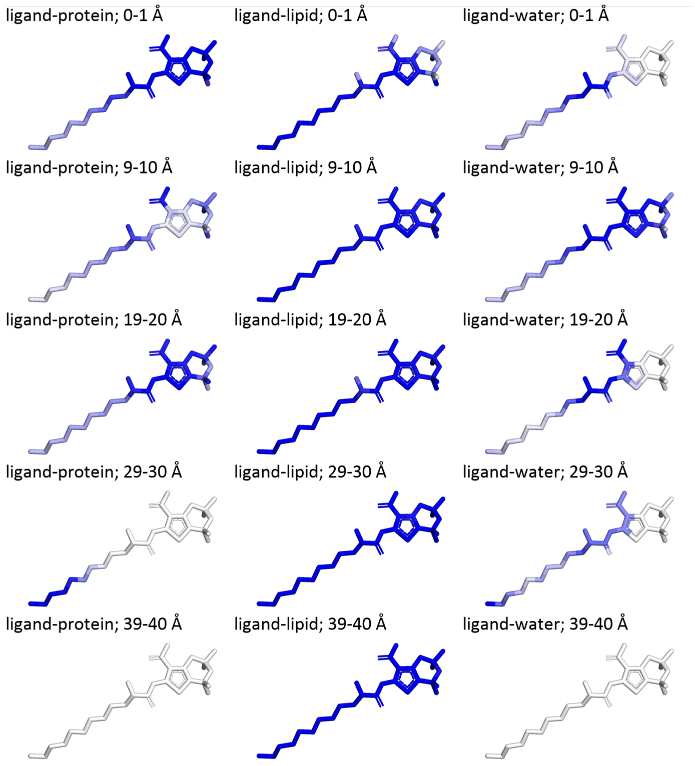

Figure S4D. Contacts from CFTRi-C10 run 2.

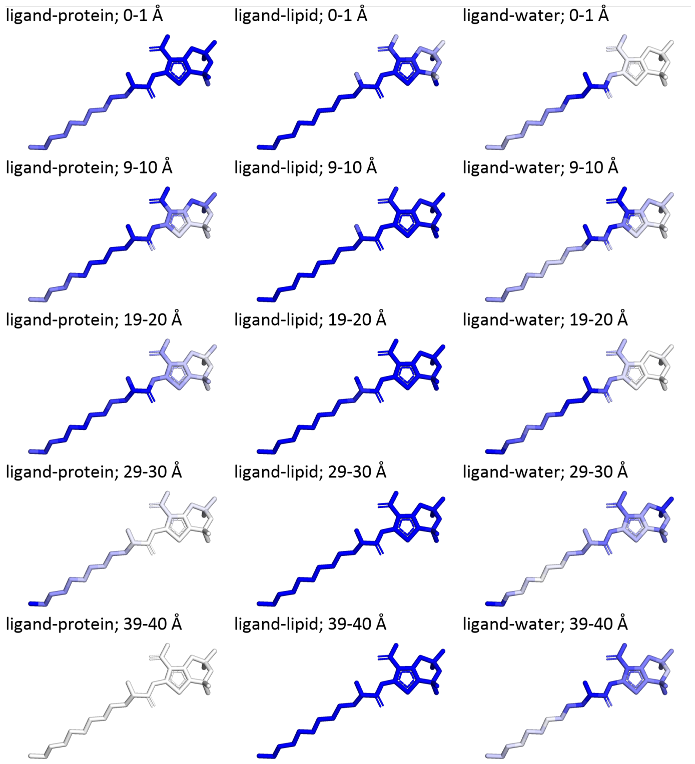

Figure S5. Ligand membrane depth (z coordinate) distributions. Data for ABBV-974 run 1 (A), run 2 (B), CFTRi-C10 run 1 (C), and run 2 (D). Plots are normalized so that the total probability in each progress coordinate (PC) bin is one (i.e. each 2D histogram bin is the probability of the ligand being at that membrane depth given its distance from the binding site). Unsampled regions are in gray. All frames from every 10th Weighted ensemble (WE) round were included, with equal weighting applied to frames within each histogram bin. Y axes are shared across rows, and x axes are shared across columns.

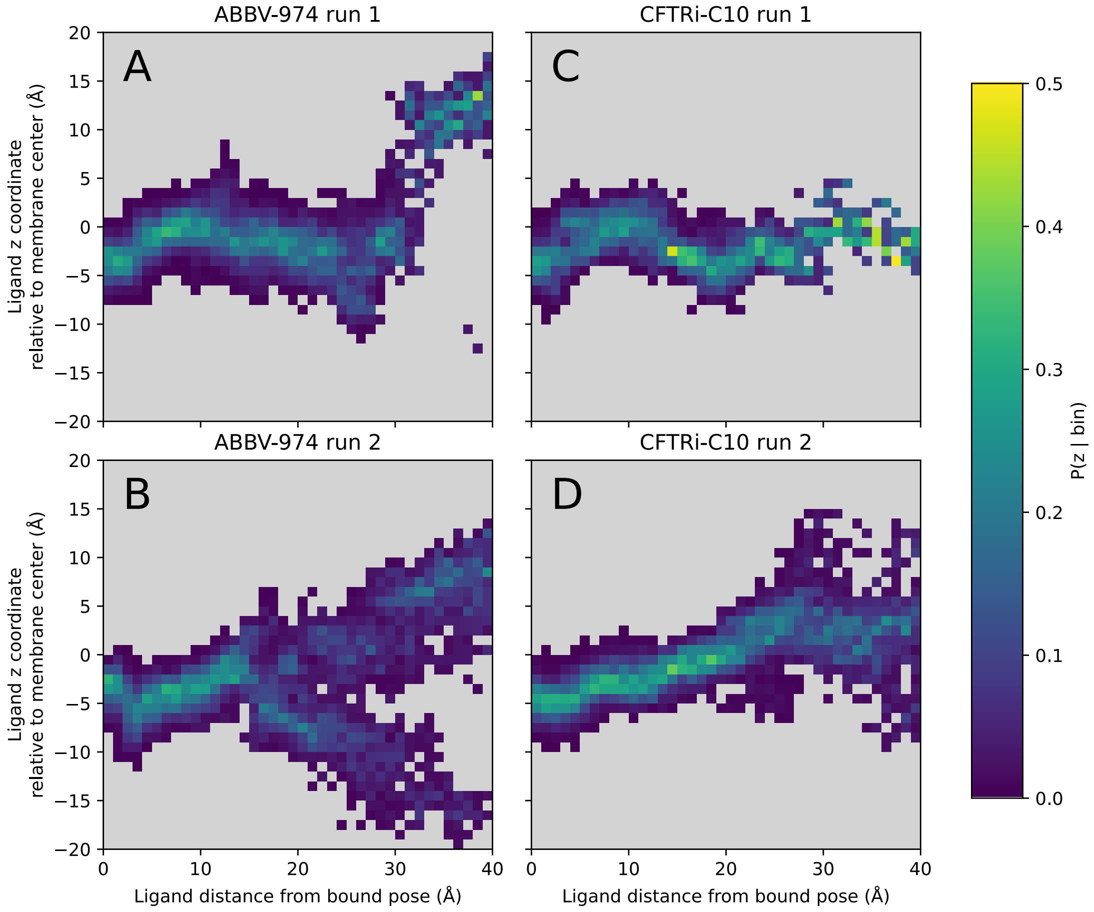

LogP Analysis of CFTRi-C3, CFTRi-C6, and CFTRi-C10

Figure S6. Calibration curve for the standard compounds displaying the equation used to determine the logP values of CFTRi-C3, C6, and C10.

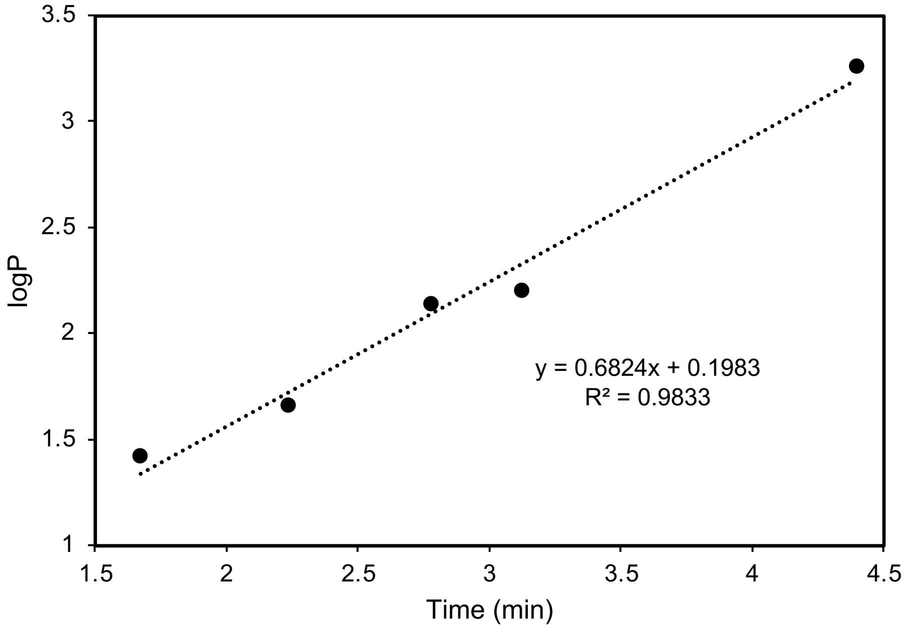

Table S1. Average retention times and logP values for the standards and compounds.

Compound	Retention Time (min)	logP
5-phenyl-2H-tetrazole	1.67 ± 0.0254	1.42
acetophenone	2.23 ± 0.0015	1.66
indole	2.78 ± 0.0025	2.14
propiophenone	3.12 ± 0.0035	2.20
valerophenone	4.39 ± 0.0080	3.26
CFTRi-C3	4.13 ± 0.0029	3.02
CFTRi-C6	5.10 ± 0.0042	3.68
CFTRi-C10	6.83 ± 0.0087	4.86

The logP values of the compounds were quantified using a procedure previously described by Valkó et. al.[1] Five standard compounds (5-phenyl-2H-tetrazole, acetophenone, indole, propiophenone, and valerophenone) were measured on an Agilent 1200 LCMS equipped with a Agilent SB-C18 2.1 x 30 mm, 3.5 um particle size column with a 1 mL/min flow rate. The solvents used were water with 0.1% formic acid and acetonitrile. Each standard sample was dissolved at 10 mM concentration in 1% acetonitrile in water, to prevent the more hydrophilic compounds from traveling

with the solvent front and ensuring more accurate time-based readings. The compounds of interest were prepared at a 1 mM concentration in 50% acetonitrile in water mixture to prevent precipitation in the column at high initial water concentrations. Each sample was injected at 1 µL. The following gradient was employed: 0-1 min, 0% acetonitrile; 1-2 min, 10% acetonitrile; 2-3 min, 20% acetonitrile; 3-4 min, 30% acetonitrile; 4-5 min, 40% acetonitrile; 5-6 min, 50% acetonitrile; 6-7 min, 60% acetonitrile; 7-8 min, 70% acetonitrile; 8-9 min, 80% acetonitrile; 9-10 min, 90% acetonitrile; 10-11 min, 100% acetonitrile. Each standard and compound sample was measured in triplicate. The logP values of the compounds were subsequently extrapolated from the calibration curve generated using the literature logP values for the standards.[1]

CFTR current decay time constants and logP values

Table S2: Experimental CFTR current decay time constants and R group logP values

Potentiator	Number of data points	Mean time constant	Time constant standard deviation	R group name	Experimental R group logP (octanol/water) from PubChem
ABBV-974	5	17.40	4.20	Pyrazole	0.26
NBD-CFTRi	5	19.90	6.50	NBD-aminopentanol	unavailable
CFTRi-C3	3	46.15	2.04	Isoamyl alcohol	1.16
JM97	2	56.50	25.51	2-phenylethanol	1.36
CFTRi-C6	3	243.02	74.81	1-heptanol	2.62
CFTRi-C10	3	2166.50	1079.90	1-undecanol	4.72

All time constant data are from this work, except for the ABBV-974 data.[2]

Table S3: Computed logP (clogP) values of potentiators and their R groups

Potentiator	clogP	R group name	R group clogP
ABBV-974	1.95	Pyrazole	0.03
NBD-CFTRi (neutral)	3.13	NBD-aminopentanol (neutral)	2.02
NBD-CFTRi (protonated)	2.06	NBD-aminopentanol (protonated)	0.41
CFTRi-C3	2.41	Isoamyl alcohol	1.33
JM97	2.65	2-phenylethanol	1.51
CFTRi-C6	3.29	1-heptanol	2.53
CFTRi-C10	4.85	1-undecanol	4.83

clogP values were calculated with ALOGPS 2.1[3]

Supplementary videos

Atoms in all supplementary videos are rendered as follows:

Lipids (and cholesterol) are not shown. Water oxygens, Na+, and Cl- are shown as red, white, and pale green spheres respectively. The protein backbone is shown as a green ribbon (or a yellow ribbon at binding site residues). CFTR residues lining the binding site (sidechains and alpha carbons only in most cases) are shown as spheres with carbons in yellow. Hydrogens are omitted with the exception of the guanidinium hydrogens on R933. The potentiator is shown as spheres with carbons in cyan. Hydrogens are omitted with the exception the polar hydrogen on the potentiator R group (the NH hydrogen on the ABBV-974 pyrazole ring or the alcohol at the base of the CFTRi-C10 acyl chain). Nitrogen atoms are in blue, hydrogen in white, and sulfur in gold. Oxygen atoms on the protein and potentiator are shown in dark red to avoid confusion with waters. Atoms further from the camera are shaded to provide depth information. Videos were rendered using PyMOL's 'smooth' function with the default settings to remove high frequency oscillations. Bond lengths and angles rendered in videos may deviate from their true values as a result of averaging by the smooth function. Each supplementary video depicts the trajectory from the Weighted Ensemble (WE) starting state to the state at which the potentiator was furthest from its bound pose (i.e. the state with the largest progress coordinate value). Note that this is a single continuous trajectory and is therefore independent of any assumptions about whether WE bins are Markovian.

Video S1: Dissociation of ABBV-974 from the CFTR binding site into the membrane headgroup region of the extracellular leaflet in ABBV-974 WE run 1. View is from within the membrane looking along the plane of the membrane. The extracellular space is up and the cytoplasm is down. The trajectory shown is 96 ns long.

Video S2: The same dissociation event shown in Video S1 viewed from a different angle. View is from the extracellular space looking down the axis of CFTR towards the cytoplasm. Extracellular waters are mostly omitted and the protein ribbons are truncated at the image plane. The interactions between the pyrazole NH group and the carboxylate of E873 depicted in Figure S3 panel A can be seen from ~18-23 seconds.

Video S3: Dissociation of CFTRi-C10 from the CFTR binding site into the membrane core in CFTRi-C10 WE run 2. View is from within the membrane looking along the plane of the membrane. The extracellular space is up and the cytoplasm is down. The trajectory shown is 99 ns long.

Video S4: The same dissociation event shown in Video S3 viewed from a different angle. View is from the extracellular space looking down the axis of CFTR towards the cytoplasm. Extracellular waters are mostly omitted and the protein ribbons are truncated at the image plane. The interactions between the R group alcohol and the carboxylate of E873 depicted in Figure S3 panel D can be seen from ~18-22 seconds.

Synthetic Details

2-(2-hydroxy-4-methylpentanamido)-5,5,7,7-tetramethyl-4,7-dihydro-5H-thieno[2,3-c]pyran-3-carboxamide **(CFTRi-C3)**

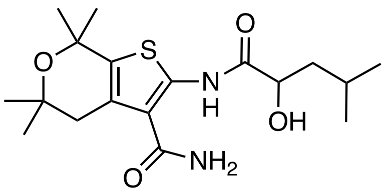

2-hydroxy-4-methylpentanoic acid (20.8 mg, 0.157 mmol, 1.0 equiv.) was dissolved in CH_2Cl_2 (1.5 mL). Acetyl chloride (24.7 mg, 0.315 mmol, 2.0 equiv.) and pyridine (24.9 mg, 0.315 mmol, 2.0 equiv.) were added at 0 °C and stirred for 1 h. The reaction mixture was diluted with water, extracted with CH_2Cl_2, dried over Na_2SO_4, filtered and concentrated under reduced pressure. The crude residue was dissolved in CH_2Cl_2 (3.0 mL). Mukuyiama reagent (60.3 mg, 0.236 mmol, 1.5 equiv.), DMAP (6.4 mg, 0.0523 mmol, 0.5 equiv.), and NEt_3 (47.7 mg, 0.471 mmol, 3.0 equiv.) were added. 2-amino-5,5,7,7-tetramethyl-4,7-dihydro-5*H*-thieno[2,3-*c*]pyran-3-carboxamide (40.0 mg, 0.157 mmol, 1.0 equiv.) was added and stirred for 16 h at 40 °C. The reaction mixture was diluted with 10% citric acid, extracted with CH_2Cl_2, dried over Na_2SO_4, filtered and concentrated under reduced pressure. The crude mixture was dissolved in THF (1.0 mL). H_2O (0.2 mL) and LiOH (2 equiv.) were added, stirred at 40 °C for 1 h, acidified with 1 M HCl, extracted with CH_2Cl_2, dried over Na_2SO_4, filtered and concentrated under reduced pressure. The crude product mixture was purified by flash column chromatography (hexanes to 50% EtOAC/hexanes) to yield **CFTRi-C3** (13.8 mg, 0.039 mmol, 25%).

^{1}H NMR (400 MHz, DMSO-d6) δ 12.02 (s, 1H), 7.51, 6.94 (br, 2H), 6.10 (d, *J* = 4.0 Hz, 2H), 4.11 – 4.07 (m, 1H), 2.69 (s, 2H), 1.79 – 1.76 (m, 1H), 1.50 - 1.47 (m, 2H), 1.43 (s, 6H), 1.17 (s, 6H), 0.89 – 0.87 (m, 6H).

LCMS: m/z calcd. for $C_{18}H_{29}N_2O_4S^+$ ($[M+H]^+$): 369.2; found: 369.2

***2-(2-hydroxyoctanamido)-5,5,7,7-tetramethyl-4,7-dihydro-5H-thieno[2,3-c]pyran-3-carboxamide* (CFTRi-C6)**

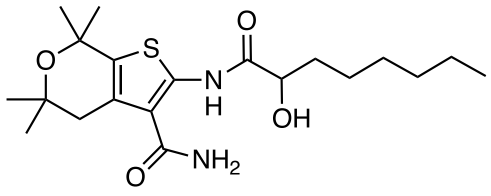

2-hydroxyoctanoic acid (25.2 mg, 0.157 mmol, 1.0 equiv.) was dissolved in CH_2Cl_2 (1.5 mL). Acetyl chloride (24.7 mg, 0.315 mmol, 2.0 equiv.) and pyridine (24.9 mg, 0.315 mmol, 2.0 equiv.) were added at 0 °C and stirred for 1 h. The reaction mixture was diluted with water, extracted with CH_2Cl_2, dried over Na_2SO_4, filtered and concentrated under reduced pressure. The crude residue was dissolved in CH_2Cl_2 (3.0 mL). Mukuyiama reagent (60.3 mg, 0.236 mmol, 1.5 equiv.), DMAP (6.4 mg, 0.0523 mmol, 0.5 equiv.), and NEt_3 (47.7 mg, 0.471 mmol, 3.0 equiv.) were added. 2-amino-5,5,7,7-tetramethyl-4,7-dihydro-5*H*-thieno[2,3-*c*]pyran-3-carboxamide (40.0 mg, 0.157 mmol, 1.0 equiv.) was added and stirred for 16 h at 40 °C. The reaction mixture was diluted with 10% citric acid, extracted with CH_2Cl_2, dried over Na_2SO_4, filtered and concentrated under reduced pressure. The crude mixture was dissolved in THF (1.0 mL). H_2O (0.2 mL) and LiOH (2 equiv.) were added, stirred at 40 °C for 1 h, acidified with 1 M HCl, extracted with CH_2Cl_2, dried over Na_2SO_4, filtered and concentrated under reduced pressure. The crude product mixture was purified by flash column chromatography (hexanes to 50% EtOAC/hexanes) to yield **CFTRi-C6** (9.2 mg, 0.023 mmol, 15%).

^{1}H NMR (400 MHz, DMSO-d6) δ 12.00 (s, 1H), 7.53 - 6.65 (br, 2H), 6.12 (d, *J* = 8.0 Hz, 1H), 4.17 – 4.07 (m, 1H), 2.70 (s, 2H), 1.70 - 1.66 (m, 1H), 1.61 – 1.55 (m, 1H), 1.43 (s, 6H), 1.27 – 1.25 (m, 8H), 1.19 (s, 6H), 0.85 (t, *J* = 8.0 Hz, 3H).

LCMS: m/z calcd. for $C_{20}H_{31}N_2O_4S^-$ ($[M-H]^-$): 395.2; found: 395.4

2-(2-hydroxydodecanamido)-5,5,7,7-tetramethyl-4,7-dihydro-5H-thieno[2,3-c]pyran-3-carboxamide **(CFTRi-C10)**

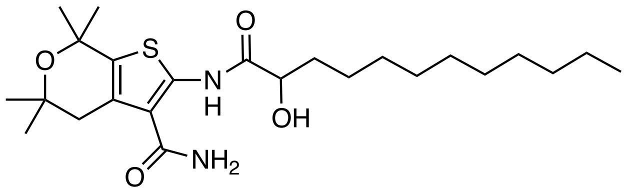

2-hydroxydodecanoic acid (34.0 mg, 0.157 mmol, 1.0 equiv.) was dissolved in CH_2Cl_2 (1.5 mL). Acetyl chloride (24.7 mg, 0.315 mmol, 2.0 equiv.) and pyridine (24.9 mg, 0.315 mmol, 2.0 equiv.) were added at 0 °C and stirred for 1 h. The reaction mixture was diluted with water, extracted with CH_2Cl_2, dried over Na_2SO_4, filtered and concentrated under reduced pressure. The crude residue was dissolved in CH_2Cl_2 (3.0 mL). Mukuyiama reagent (60.3 mg, 0.236 mmol, 1.5 equiv.), DMAP (6.4 mg, 0.0523 mmol, 0.5 equiv.), and NEt_3 (47.7 mg, 0.471 mmol, 3.0 equiv.) were added. 2-amino-5,5,7,7-tetramethyl-4,7-dihydro-5*H*-thieno[2,3-*c*]pyran-3-carboxamide (40.0 mg, 0.157 mmol, 1.0 equiv.) was added and stirred for 16 h at 40 °C. The reaction mixture was diluted with 10% citric acid, extracted with CH_2Cl_2, dried over Na_2SO_4, filtered and concentrated under reduced pressure. The crude mixture was dissolved in THF (1.0 mL). H_2O (0.2 mL) and LiOH (2 equiv.) were added, stirred at 40 °C for 1 h, acidified with 1 M HCl, extracted with CH_2Cl_2, dried over Na_2SO_4, filtered and concentrated under reduced pressure. The crude product mixture was purified by flash column chromatography (hexanes to 50% EtOAC/hexanes) to yield **CFTRi-C10** (14.0 mg, 0.031 mmol, 20%).

^{1}H NMR (400 MHz, DMSO-d6) δ 11.98 (s, 1H), 7.51, 6.94 (br, 2H), 6.10 (d, *J* = 4.0 Hz, 1H), 4.19 – 4.05 (m, 1H), 2.68 (s, 2H), 1.73 – 1.56 (m, 2H), 1.41 (s, 6H), 1.24 -1.19 (m, 16H), 1.17 (s, 6H), 0.83 (t, *J* = 8.0 Hz, 3H).

LCMS: m/z calcd. for $C_{24}H_{39}N_2O_4S^-$ ($[M-H]^-$): 451.3; found: 451.4

References

(1) Valkó, K.; Bevan, C.; Reynolds, D. Chromatographic Hydrophobicity Index by Fast-Gradient RP-HPLC: A High-Throughput Alternative to Log P/Log D. *Anal. Chem.* **1997**, *69* (11), 2022–2029. https://doi.org/10.1021/ac961242d.

(2) Yeh, H.-I.; Qiu, L.; Sohma, Y.; Conrath, K.; Zou, X.; Hwang, T.-C. Identifying the Molecular Target Sites for CFTR Potentiators GLPG1837 and VX-770. *J. Gen. Physiol.* **2019**, *151* (7), 912–928. https://doi.org/10.1085/jgp.201912360.

(3) Tetko, I. V.; Gasteiger, J.; Todeschini, R.; Mauri, A.; Livingstone, D.; Ertl, P.; Palyulin, V. A.; Radchenko, E. V.; Zefirov, N. S.; Makarenko, A. S.; Tanchuk, V. Y.; Prokopenko, V. V. Virtual Computational Chemistry Laboratory--Design and Description. *J. Comput. Aided Mol. Des.* **2005**, *19* (6), 453–463. https://doi.org/10.1007/s10822-005-8694-y.
